# A systematic scoping review identifying effective virtual reality stress tasks inducing physiological reactivity for future wearables validation

**DOI:** 10.3758/s13428-026-03161-3

**Published:** 2026-09-18

**Authors:** Magdalena Sikora, Xiaochang Zhao, Jan-Willem van ’t Klooster, Zeynep Koyuncu, Eco de Geus, Matthijs Noordzij

**Affiliations:** 1 Department of Psychology, Health and Technology, University of Twente, Enschede, The Netherlands; 2 Department of Biological Psychology, VU Amsterdam, Amsterdam, The Netherlands; 3 Amsterdam Public Health research institute, Amsterdam UMC, Amsterdam, The Netherlands

**Keywords:** Wearable, Validation, Virtual reality, Stress induction, Psychophysiology

## Abstract

Efficient and ecologically sound validation of wearables remains a major challenge in stress research. Virtual reality (VR) could support this by providing standardized, immersive stress induction, but the field lacks a comprehensive and exhaustive overview of which VR tasks reliably evoke physiological stress responses. This review delivers a systematic comparison of VR stressors, mapping 11 tasks into three core stressor categories (social-evaluative, environmental, and cognitive) and offers a synthesis of their effectiveness, methodological characteristics, and suitability for VR-based wearables validation. We examined VR’s effectiveness for physiological stress elicitation, both qualitatively with the focus on task design and quantitatively through available effect sizes of responses. We observed high heterogeneity in stressor design and measurement methods, with the VR Trier Social Stress Test (VR-TSST) and the high-altitude task emerging as most promising VR stressors. Although several studies incorporated wearables, proving feasibility of such setups, none used VR explicitly for validating them. This review supports the use of VR for stress elicitation and for validation of wearable devices. It also enables informed stressor selection and VR study design while highlighting the existing gaps and future directions.

**Supplementary Information:**

The online version contains supplementary material available at https://doi.org/10.3758/s13428-026-03161-3.

## Introduction

In recent years, both wearable devices and virtual reality (VR) have positioned themselves as useful and increasingly utilized stress research tools. Wearables, such as smartwatches or rings, can be used to continuously and non-invasively capture stress-relevant physiological measures of the autonomic nervous system (ANS), such as heart rate (HR) or electrodermal activity (EDA). The high usability of wearables combined with the unobtrusive and multimodal sensing capabilities offers great opportunities for longitudinal, ambulatory assessment of psychophysiological changes (Beukenhorst et al., 2020; Kleckner et al., 2021). In parallel, VR has emerged as a valuable platform for standardized, immersive stressor presentation (Van Dammen et al., 2022), where physiological measures are increasingly incorporated to quantify the effects of virtual stimuli and interactions (Halbig & Latoschik, 2021). These measures are most commonly obtained through gold-standard lab-based recording, such as electrocardiography (ECG), with some researchers opting to utilize wearables (Halbig & Latoschik, 2021). Yet, the high usability introduced by wearable devices often comes at the cost of their accuracy (de Geus & Gevonden, 2024). Knowing which wearables to rely on in terms of validity, particularly for capturing physiological (stress) responses, remains a challenge. The quick development of the wearables market, including rapid turnover of devices, calls for continuous and efficient validation of promising wearables (Schoenmakers et al., 2025; van Lier et al., 2020). Where the current validation efforts fall short—for example, low ecological validity or cumbersome and labour-intensive setups—VR offers a promising opportunity through creation of immersive, naturalistic, yet highly controllable stressors. With the growing reliance on wearables by researchers and with VR’s ability to evoke physiological reactivity (Halbig & Latoschik, 2021; Helminen et al., 2019, 2021; Van Dammen et al., 2022) while maintaining high experimental control, we believe these two fields could further be integrated into a VR-based stress induction protocol for validating wearables. To our knowledge, supported by our previous work on wearables validity (Schoenmakers et al., 2025), VR has not yet been used in this context. Given the rapid development and high variability of technologies and methods involved in both VR and wearables application to stress research, we believe a scoping review is the most appropriate method to map the existing evidence relevant to combine these two fields. Therefore, this review aims to gain insights into the design and effectiveness of existing VR stress paradigms for eliciting stress reactivity, thereby examining their potential for inclusion in validation protocols and guiding the development of VR-based methods for testing wearable devices.

### Rationale for incorporating VR in wearables validation

At its core, validating wearables in a laboratory setting requires the following two aspects to be met: (1) an option to, in a controlled manner, subject a person wearing a device to different conditions in a way that results in sufficient, detectable, and relevant variations in physiology, and (2) a possibility to capture these changes with both a wearable device and gold-standard recording. The combination of these two aspects allows us to assess devices on the level of *construct* validity (e.g., heart rate captured by a wearable goes up as a result of stress induction, differentiating the stressful experience from a neutral baseline), as well as on the level of *convergent* validity (e.g., the degree to which the wearable heart rate data converge with those of a gold-standard reference device). While we believe that both of these prerequisites can be met while utilizing VR stress induction for validating wearables, it remains unclear to what extent these points can be satisfied. Therefore, this review explores which tasks and their characteristics produce such variation at a sufficient level, and whether both wearables and gold-standard recording is easily implementable in VR studies, ideally within the same experimental setup.

This review also investigates whether and how VR can benefit laboratory-based validation protocols, currently suffering from two main factors hampering their efficiency and output: operational inefficiency and the potential disconnect between laboratory, artificial conditions and the dynamics of real life, often referred to as low ecological validity. Both of these shortcomings could potentially benefit from the introduction of VR as a tool for easily reproducible, standardized, and more naturalistic validation.

Firstly, existing lab-based validation studies, while rigorous, can be cumbersome and labour-intensive to set up and execute. This hampers the speed of validation and makes it challenging to repeat. These protocols are often quite complex, with up to 26 experimental conditions (van der Mee et al., 2021) including postural changes, controlled physical activity, simulated daily activities, and, less commonly, stress tests (Menghini et al., 2019; van Lier et al., 2020). As such, they require substantial effort in terms of task preparation and execution. For example, one of the most established lab-based stressors, the Trier Social Stress Test (Kirschbaum et al., 1993), involves giving a 5-min speech to a panel of three trained judges who always have to be present. The task also introduces a need for multiple facilities. A recent umbrella review (Doherty et al., 2024) shows that the current validation practices represent only 3.5% of potential validation. This speaks to how difficult it is to keep pace with the rapidly expanding field of wearables. Translating the lab-based protocols to VR and making the tasks self-contained in terms of instructions and execution may lower the high experimental demands and positively impact the operational efficiency, scalability, and durability of a validation pipeline. This in turn could allow for more devices to be tested in an efficient and resource-effective manner over extended periods and at a faster pace. Such a VR validation setup could also easily be transported, or reproduced at another distant facility, aligning well with the portable nature of wearables. Ideally, such a stand-alone virtual application could easily be shared with other researchers or industry partners looking for a standardized way of assessing wearable devices.

A second aspect which undermines the lab-based validation is that it tends to fall short in addressing the ecological validity, making the findings less applicable to daily-life use. In the context of validating wearables for stress research, ecological validity can be considered the degree to which the dynamic, rich contextual factors of stressful experiences are preserved (Epel et al., 2018; Hoemann et al., 2020). According to Hoemann et al. (2020), daily life emotional processes, such as experience of stress, depend highly on situational and contextual factors. The way these experiences are constructed and how they manifest also vary strongly between and within persons. This richness of experience therefore cannot be recreated in a single laboratory session with artificial conditions. Yet, existing validity studies assess the devices mainly through such highly controlled lab protocols, often not including any mental stressors, and instead focusing strongly on postural changes and physical activity of different levels (Agostinelli et al., 2023; Blok et al., 2021; Cosoli et al., 2023; Hajj-Boutros et al., 2023; Jayasekera et al., 2023; Nazari & MacDermid, 2020; Schaffarczyk et al., 2022; Støve et al., 2023; Stuyck et al., 2022). Recent recommendations (Kleckner et al., 2021; Mühlen et al., 2021) recognize that wearables should be validated in conditions and context representative of their intended use. However, while ambulatory measurement is highly relevant for understanding the range of stress experiences and how these unfold in daily life, the heterogeneity of these processes makes pure ambulatory validation of wearables challenging. While beneficial for more precise estimation of actual quality of incoming data, ambulatory validation would still suffer from the lack of experimental control, especially as related to a likely absence of acute stressful events in the selected observation period or their unclear temporal trajectories (Epel et al., 2018). Can et al. (2020) additionally show that wearables data from laboratory stress-inducing protocols can improve stress detection algorithms once deployed in daily life, higlighting the value of controlled stress induction.

We hence argue that VR may form a bridging platform between laboratory and ambulatory validation, where the experimental control is met with an option to increase ecological validity through a range of immersive scenarios that are richer in contextual factors and characteristics relevant to construction of stressful experiences. While such validation does not enable us to increase our understanding of dynamic, real-world stress-related processes, it is in our view a necessary first step in establishing whether a device can even be used for such a purpose—that is, whether it can accurately capture the changes in physiological signals related to experiencing acute stress. Therefore, validation protocols would benefit from including psychological stressors, not only of established validity in producing a physiological stress response (Kleckner et al., 2021), but also designed to represent the range and complexity of real-world, context-dependent stress construction. The degree to which VR may support this aim is one of the driving questions of this review.

### Value of VR for stress induction

Next to high reliability and reproducibility common for lab-based studies, VR offers other advantages, such as increased flexibility in experimental design and manipulation of its components, or the potential to expose participants to scenarios that are impossible, unsafe, or unethical to replicate in a lab setting, for example, when targeting the fear of heights or certain phobias. This includes tailoring the stress tasks to investigate physiological reactivity in specific populations (e.g., job emergency simulations for occupational research, or crowded environments for social anxiety). The flexibility in stimuli design and presentation, and being able to transport participants to (a series of) different virtual environments without them leaving the lab, opens up doors to various possible designs. However, such flexibility of VR and the broad application of it for stress elicitation results in high variability of study designs, stressor tasks, and elements, as well as various stress operationalizations and measurement methods.

Despite the growing interest in the use of VR for stress induction, a synthesis of evidence on task characteristics and their types and effectiveness appears to be lacking. Most existing reviews on VR and stress focus on stress reduction and not induction (see for example Abdullah et al., 2021; Gentile et al., 2023; Ladakis et al., 2021; Lüddecke & Felnhofer, 2022). We identified a single systematic review focusing on stress reactivity in VR (Van Dammen et al., 2022), which, through a meta-analytic approach, provides support for the ability of VR to evoke stress responses across many physiological biomarkers. While the findings of this study confirm that VR-TSST produces a higher magnitude of HR and EDA responses than other tasks, it remains unclear exactly what the other VR stressors are, how they differ, and, most importantly, which of them hold promise for VR stress induction. Similarly, while VR-TSST has shown comparable reactivity to its in vivo counterpart (Helminen et al., 2021), it remains unknown how other VR simulations compare to their lab-based versions. While theoretical guidelines on lab-based acute stress induction already exist (e.g., on importance of uncontrollability and social evaluation for cortisol responses; Dickerson & Kemeny, 2004), it remains unclear to what extent the VR stressors are positioned within such theoretical reasoning, and whether the strong pragmatic focus on advantages of VR takes away from theoretically guided stressor design.

### Potential limitations of VR for stress induction and validating wearables

While promising, the use of VR is not without challenges. Technological limitations, such as motion sickness and nausea experienced by some participants, may affect data quality and limit the generalizability of findings, particularly in studies requiring prolonged exposure to virtual environments. Prolonged exposure also becomes an issue when faced with the need for validation protocols to cover a variety of stressor characteristics and contexts in hopes of increasing their ecological validity. This in turn closely connects to the effect VR itself may have on physiological signals relevant for validating wearables, not only through potential cybersickness, but also through aspects such as VR’s novelty. Additionally, VR as an artificial representation of the real world results in different levels of immersion and presence. Such sense of “being there” often depends on various factors, such as the VR technology used or the level of realism in the VR scenario, including spatial, social, and embodiment aspects (Bareišytė et al., 2024). This indicates that immersion or presence should not be equated with ecological validity and that the relationship between the design of the VR stressor task, the way it is presented, and the resulting subjective and physiological reactions are complex. Unwinding this complexity through a structured overview of VR stress tasks, their characteristics, and the resulting reactivity may help researchers to better understand which VR tasks and their features are relevant for effective and targeted stress induction. Lastly, despite the increased use of VR, the effort required to create VR scenarios is substantial, and the availability of up-to-date, research-suitable VR applications remains a challenge, as not all commercially available solutions meet the necessary standards for scientific validity and experimental control. Building towards stable scientific open-access tools for VR stress research could additionally benefit the standardization and range of possibilities within this varied field.

### Current study

This review is driven by the promising but so far unexplored consolidation of these two converging yet mostly separate fields: wearables validation and VR stress induction. Since it remains unknown whether VR has previously been used for validating wearables, and given the need for further synthesis of existing VR stress induction research, this review aims to scope both of these research areas and map the evidence relevant for their harmonization. To further support the development of a VR-based validation pipeline, we aim to understand current VR stress research practices and motivations, the types of stress tasks implemented in VR, and the types of stressors and stress measures most commonly used, with the greatest focus on task effectiveness for physiological reactivity. The primary research questions that this review aims to address are as follows:

- RQ1: How has VR been utilized for validation of wearable devices measuring physiological signals, and what are the characteristics of these studies in terms of tasks, methods, and physiological parameters?
- RQ2: What are the currently established VR tasks used to induce stress in research settings and what type of stressors do they use (e.g., social-evaluative threat, cognitive elements)?
- RQ3: What is the reported effectiveness of different VR stress tests in producing a stress response and how is it measured (e.g., comparison with self-reported stress, comparison with the in vivo lab condition)?
- RQ4: How is the choice for a given VR stress induction test motivated with regard to both theory (e.g., based on a particular stress model) and practical reasons (e.g., a VR version of the task is already available, previously confirmed effectiveness of a given task)?

Moreover, this review aims to explore which existing VR methods could be readily employed in a validation pipeline, and has as a secondary research question:

- RQ5: Are any of the identified VR tasks freely available as open-source software and implementable in a standard psychophysiology laboratory?

Finding answers to the questions above will map the current state of the stress induction research in VR. Finally, the outcomes of this review will inform the subsequent development of the VR validation pipeline for wearable devices.

## Methods

### Protocol and registration

The protocol for this scoping review was developed in accordance with the Preferred Reporting Items for Systematic reviews and Meta-Analyses extension for Scoping Reviews (Tricco et al., 2018), and its structure was based on the Generalized Systematic Review Registration Form (van den Akker et al., 2023). The protocol was created under supervision of an information specialist and was preregistered on OSF on 21 March 2024 (https://osf.io/sp5z8).

### Eligibility criteria

For a study to be eligible for this review it needed to fulfil the following set of inclusion criteria. Papers were included if VR was used to induce stress with the help of a stress-inducing simulation or task and if healthy human participants were included (also if present only as a control group). In order to maximize the scope of the search, next to peer-reviewed articles, conference proceedings and PhD dissertations were deemed eligible for inclusion. Papers had to be written in English, and, seeing how VR is a considerably modern technology, no restrictions were specified for publication date. Additionally, papers were included if VR and in vivo stress induction was compared. However, if VR was used to induce stress for training purposes, e.g., simulation training for soldiers, such papers were excluded for two reasons. Firstly, such stress-inducing job-specific tasks require more challenging setups (e.g., large spaces and dedicated, specialized equipment needed for simulated police, army, firefighting, or surgeon training to name a few). Secondly, such simulation training is profession-based and, as such, the task used is most likely skill- or knowledge-specific. Due to the low potential for reproducibility and generalizability of such tasks, VR simulation training papers were therefore excluded. Moreover, studies were excluded if they used VR to influence stress level but the stressor itself was administered outside of VR, e.g., via a real-life stressor before or after placing the participant in VR. We have limited the term VR to apply to either head-mounted displays (HMDs) or cave automatic virtual environment (CAVE)-like stereoscopic room systems (Cruz-Neira et al., 1993). Studies that used the term VR or virtual environment to refer to a regular screen stimuli were excluded, with the exception of driving simulators, where a screen projection (curved and ultrawide) is sometimes used. In the case of validation papers, VR had to be used to validate wearable devices that can measure physiological responses, assessing either the reliability or convergent validity of the provided signals. This resulted in an exclusion criterion for validation of devices that were simply a VR-related tool, such as HMDs themselves, haptic feedback devices, tactile feedback gloves, or motion capture systems. Moreover, if a study simply used a wearable device to measure physiological signals, including those relevant to the stress response, but with no assessment of its reliability or convergent validity, it was not seen as a validation study and was consequently excluded, unless a VR stress induction task was present. Similarly, VR studies that used wearables to collect physiological signals for building classification algorithms (e.g., emotion detection) were excluded since the accuracy of such algorithms does not necessarily equate to accuracy of the signals and is therefore not sufficient evidence of the device’s validity. Lastly, other reviews and meta-analyses were excluded to ensure clear mapping of the existing primary evidence.

### Search strategy

Five databases were used to widen the scope of the search: ACM Digital Library, IEEE Xplore, PubMed, Web of Science, and Scopus. While ACM Digital Library and IEEE Xplore were chosen as technology-rich sources, PubMed was used to scope publications of a medical nature, with Web of Science and Scopus serving as large, multidisciplinary resources. The search strategy was developed iteratively and under guidance from an information specialist (see preregistration; https://osf.io/sp5z8). The final keyword string was a combination of three distinct components—VR itself, stress induction, and validation of wearables—and was furthermore slightly adjusted to match the needs of a given database. The initial search which guided the development of the search strategy was performed twice, in August and October 2023 and again in January and February of 2024. This search revealed for example an unproportionately high number of results for ACM DL (9,046 papers) and Scopus (33,543 papers). After screening the first 50 abstracts and determining a very low relevance rate, the *abstract* limiter was used for those two databases, consequently greatly increasing the relevance rate of papers identified. Once the strategy was finalized, the final search took place on 8 March 2024 (detailed search log included as Supplementary 1). Next to the database search, backward reference tracking was planned to be performed only if the source was not already identified and clearly referenced in-text in another paper as a VR stress induction study.

### Screening

Initially, pilot screening was conducted to refine the screening process and make adjustments to the eligibility criteria. For example, the irrelevance of studies using wearables in VR for classification algorithms was established thanks to this initial phase. Subsequently, the screening phase was divided into the two regular phases of titles and abstracts, followed by full-text screening. All the screening was performed independently by two reviewers (M.S., X.Z.), with a third reviewer performing a quality check during the titles and abstract screening phase (J.W.v.K.) Conflicts between reviewers were resolved through discussion and, when needed, by involvement of all other authors.

To facilitate the screening phase, the records identified among all the databases were combined into a single EndNote (RRID:SCR_014001) library. This reference manager was used to remove duplicates and to ensure the presence of all the abstracts. The data were then imported into ASReview software (https://asreview.nl/), and the titles and abstracts were screened using their active learning method (https://asreview.nl/blog/active-learning-explained/). In our previous work (Schoenmakers et al., 2025), this method decreased the time needed to identify the relevant records, as it uses the decisions made by the researcher (relevant/irrelevant) to determine the next, most likely relevant record to suggest to the reviewer. The algorithm therefore leads to a constant reordering of the papers to be screened based on their potential relevance, where it is assumed that the relevant papers will be identified faster than in a completely random set. However, it remains unclear when the screening can be stopped, and several stopping rules have been developed to guide this process. For this study, the SAFE procedure (Boetje & van de Schoot, 2024) was employed, as it appears to combine the strengths of different current approaches (relevant calculations present in Supplementary 1).

However, ASReview, with all its strengths, does not facilitate simultaneous screening by multiple reviewers. Therefore, two reviewers (M.S., X.Z.) performed the initial ASReview screening separately (with a third independent reviewer involved in the last phase of the SAFE procedure). The downloaded results were then merged into a single Excel file using Python, where the decisions on the relevance of the papers from both sets were checked for conflicts (resolved through discussion) and which was used to calculate inter-rater reliability (Cohen’s κ =.977). The final set of relevant papers together with their full texts was then imported into Covidence software (RRID:SCR_016484), which was used to facilitate the full-text screening stage. Conflicts were identified by the software and resolved again through discussion between reviewers. Subsequently, Covidence was also used to support data extraction from the final set of relevant papers.

### Data extraction

A data charting form was developed as part of the protocol for this review, but was iteratively updated in the first phase of the extraction phase. Two reviewers (M.S., X.Z.) independently charted the data on a subset of five papers to iteratively finalize the form, which was then also uploaded to the OSF page. The final extraction form template was created in Covidence (see Supplementary 2). Next to metadata, we extracted information on study characteristics (e.g., population type, sample size), the stress task(s) used (e.g., task description, types of stressors targeted), methods of assessing the stress response (e.g., subjective assessment, details of physiological assessment), rationale for selecting a given stress test (theory-based or pragmatic), technologies used (e.g., type of VR system, wearable devices included), and results and conclusions in a form that would facilitate synthesis of the reported effectiveness of the different tasks. For this reason, a list of physiological parameters relevant to the ANS activity within the stress response was created (see Appendix Table 4) based on recommendations of de Geus and Gevonden (2024) and was extended to include cortisol and α-amylase as stress-relevant hormones. During data extraction, we observed a great variety on multiple relevant levels. While most studies focused only on statistically significant variations, some attempted to quantify these effects. In both cases, these quantifications were performed on different levels, as relevant to a given study design and aims, and not necessarily through the baseline-to-peak analysis. Although the baseline-to-peak approach was present at times, often it was replaced with a pre-to-post stressor assessment. Additionally, some authors focused on stress induction differences between conditions, others on time effects of the exposure, and sometimes even on stressor-recovery differences. We completely omitted results if only the stressor-recovery data were present with no pre-stress assessment, as it seems that those would speak more to the effectiveness of the recovery stimuli. Considering this high variety in reporting, we took a categorical approach that would still allow effectiveness comparisons. The effectiveness of tasks was classified as small, moderate, or large based on standard thresholds for common effect size metrics (see Table 1, values based on Cohen, 1988; Nieminen, 2022; Peterson & Brown, 2005). Where available, *p*-values indicating significant changes from baseline to stress were also noted to establish the presence or absence of effects.

**Table 1:** Effect size thresholds for categorizing the reported effectiveness of tasks

Effect size	Correlation (*r*)	Hedges' *g*/Cohen’s *d*/SMD	Standardized β	η^2^ (ANOVA)	η^2^ (regression)
Small	0.10–0.29	0.20–0.49	0.05–0.24	0.01–0.05	0.02–0.12
Moderate	0.30–0.49	0.50–0.79	0.25–0.44	0.06–0.13	0.13–0.25
Large	≥ 0.50	≥ 0.80	≥ 0.45	≥ 0.14	≥ 0.26

Only data reported in the original publications were used, with no additional information being requested from authors. Given the scoping nature of this review, no risk of bias assessment or further statistical synthesis was conducted. The first author (M.S.) performed an additional quality check on the exported data and inputted missing or unclear information. The final dataset was then used for further synthesis.

### Synthesis

The extracted data were first aggregated (e.g., how many studies used the VR version of the Trier Social Stress Test) with regard to study characteristics, stress tests identified, types of stressors these tasks targeted, number of studies that used VR to validate wearable devices and the validation characteristics, technologies used, and ratio of well-established versus novel stress tests, and the observed advantages and disadvantages of in vivo versus VR stress tests were summarized. The data that pertain to effectiveness of a VR task in producing a stress response were analysed through grouping based on a task included, quantitative categorization, and their reported effect sizes.

During this aggregation process, studies were grouped into emerging task categories to better characterize task design and the dominant stressors implemented. This categorization was partly inductive, as specific categories emerged from the included studies, but was also theoretically guided by relevant existing work (Dickerson & Kemeny, 2004; Epel et al., 2018; Kirschbaum et al., 1993; Richter et al., 2008; Woody et al., 2018) specifying that acute stress often emerges from uncontrollability and demands imposed by (1) *social-evaluative threat* (feeling judged by other), (2) physical threat or discomfort arising from interaction with one’s *environment*, or (3) extensive *cognitive* demands related to a task’s difficulty. These three aspects were chosen to classify the stress task categories identified based on their *content*—*what* a stressor is, not *how* it is structured, perceived, and appraised. For example, novelty or uncontrollability can be introduced into different stressor categories as an additional, simultaneously coexisting context layer and offer little descriptive specificity when aiming to categorize VR stress tasks into distinct types. While social-evaluative elements, environmental threat, and cognitive challenges may still easily coexist within a task, they have clearer descriptive boundaries. Since tasks often combine multiple stressor elements, the classification was based on the primary type of stressor used. For example, VR-TSST, having to perform in front of judges, was classified under the *social-evaluative threat* as the primary stressor type, despite including a cognitive, serial subtraction math task in its design. This was based on the fact that this task primarily elicits the perception of being judged by others, first on the speech delivered and then on the mistakes made during the cognitive task.

Tasks were hence classified under *social-evaluative threat* if the primary source of the stressor was the threat to one’s self-identity through judgement and negative social cues from others. This includes tasks with an audience judging a person on their ability and performance in tasks such as public speaking or interviews, but also task categories in which a person was socially excluded or felt threatened by the social context, such as in the presence of hostile virtual avatars. Similarly, if it was the environment that was the greatest source of stressful experience through an imposed threat to one’s physical self, the task was classified as primarily *environmental.* Examples here include tasks utilizing virtual heights, emergency scenarios such as explosions, fires, or floods, or exposure to horror-like environments and events. *Cognitive* tasks were classified as ones in which the cognitive, mental demands were dominant, for example, the Color-Word Stroop Test (Stroop, 1935) and memory or spatial-navigation tasks. As already mentioned, many stress tasks combine different stressor elements. These were hence listed as secondary sources of stress for each specific type of a task categorized, along a detailed description of what the task category entails and what its characteristics are. When relevant, following Epel et al. (2018), we additionally specified whether stressor tasks required clear active or passive engagement.

The task categories that emerged were then explored on their stress-inducing effectiveness. To support the interpretation of task effectiveness from the varied sources of evidence, we followed the visual synthesis methodology first proposed by Ogilvie et al. (2008) where the so-called harvest plot is used to visualize the data. Harvest plots enable the visualization of the direction and relative strength of effects across studies, even when data cannot be expressed in uniform statistical metrics. Thus, they offer a transparent way to compare whether VR stress tasks elicit physiological reactivity while making explicit the diversity in study designs and reporting. We utilized a vector graphics software program (Adobe Illustrator (RRID:SCR_010279)) to create this plot with pixels as a standardization unit to represent the sample size of the study, e.g., bar height of 42 pixels corresponding to a sample size of 42 participants. The effectiveness insights were also treated qualitatively, where general conclusions were drawn in relation to task design and for the available in vivo comparisons. Additionally, other qualitative components (e.g., reasons mentioned for selecting a specific task) were synthesized qualitatively and presented narratively. Finally, the availability of the VR stress tasks was listed together with the conditions of their use if applicable.

## Results

**Fig. 1:**
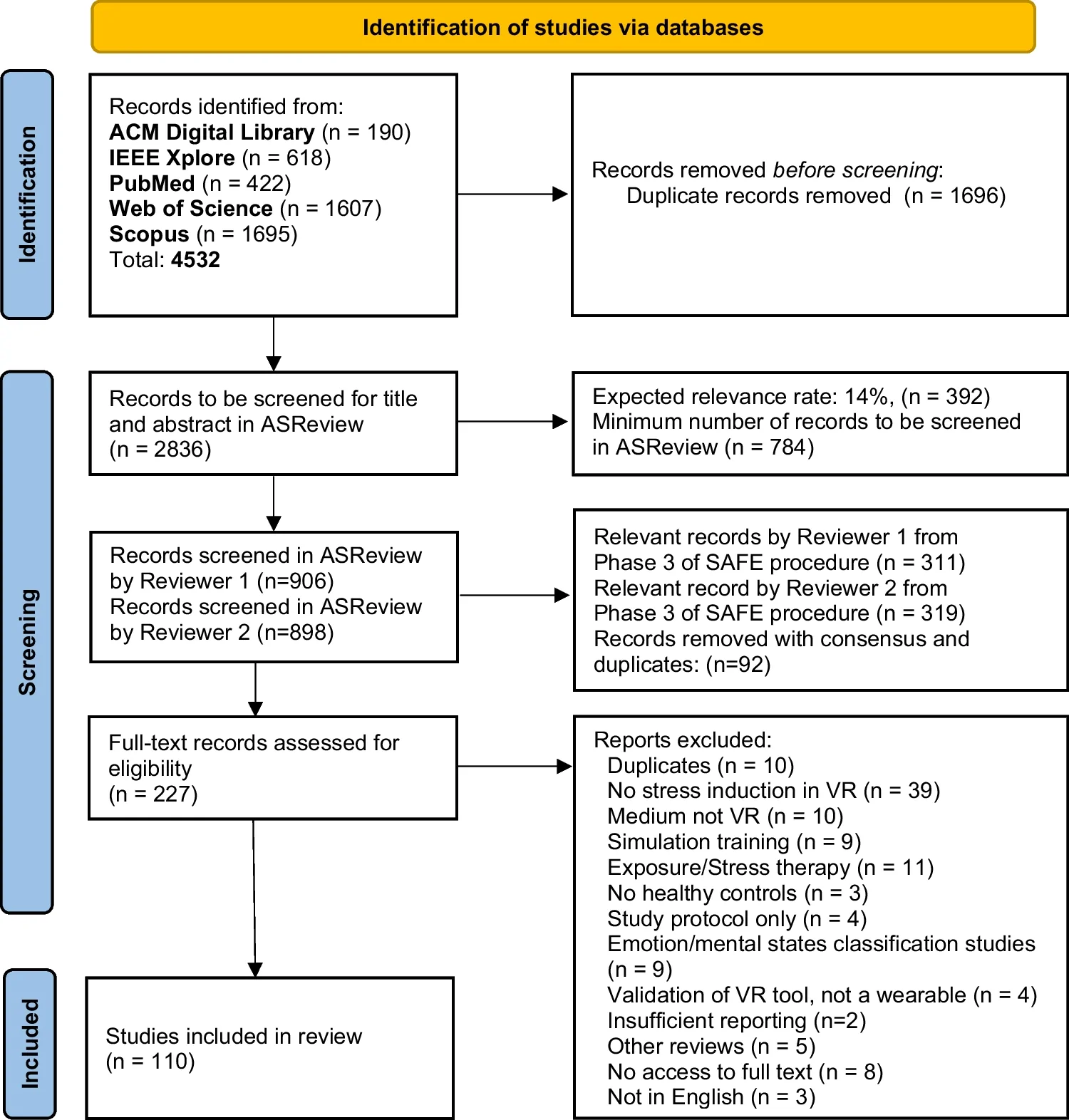
PRISMA flow diagram illustrating the selection process for studies included in this review

The number of records identified, screened, and removed together with the reasons for their exclusion can be seen in Fig. 1.

### Study characteristics

#### General overview

A wide range of studies (*N* = 110) were included in this review (see Table 2 for an example overview of six studies and Appendix Table 5 for the full table). Studies reviewed were published between 1997 (Turner et al., 1997; early driving simulator) and 2024, with most targeting a general healthy adult or student population (94/110, 85%), followed by psychopathology samples with accompanied healthy controls, including psychosis (*n* = 6), anxiety (*n* = 2), panic disorder, agoraphobia, acrophobia, work stress-related exhaustion disorder, and burnout (all *n* = 1). There were also studies focused on somatic illnesses or symptoms: patients with primary focal hyperhidrosis, obese individuals, and smokers (all *n* = 1 and with control groups). The included studies covered a wide range of ages (15–82 years), with a pooled sample size of 5,967 individuals and with sample sizes ranging from 2 for the smallest-size pilot assessment (Mevlevioğlu et al., 2022) to 735 as part of the Cardiovascular Twin cohort (Wang et al., 2009). Several studies, all focusing on hormonal secretion, included only male (*N* = 17) or only female (*N* = 3) samples to separate gender-based effects. While 17% of studies (*n* = 19) recorded only the physiological stress response, 11% focused purely on subjective stress reports (*n* = 12), while 72% investigated both concurrently (*n* = 79). Physiology was most commonly recorded with lab-based equipment (*N* = 68, omitting saliva testing), with Biopac (*N* = 11), Brain Products (*n* = 5), and Thought Technology (*n* = 5) systems being used most often. No studies were identified that utilized VR to validate wearable devices, but 12 studies used wearables to obtain the signals of interest, with Empatica E4 (*n* = 4) and Polar H10 (*n* = 3) being most common. Since most data were recorded by gold-standard lab systems and at high sampling rates, it can be assumed that the information gathered is valid for drawing an initial inference on the task effectiveness. It should be noted that for many studies, assessing the task effectiveness as reflected by physiological changes was not a goal in itself. Instead, the response to a stressor was recorded to support the primary research questions, such as investigating the association of hair cortisol and the stress response and recovery (Heming et al., 2024). Lastly, 18 records compared the VR stress task to its in vivo laboratory counterpart, allowing for an assessment of the comparability and validity of VR-based stress induction.

**Table 2:** Characteristics of a subset of studies included in this review representative of the emerging categories of stress tasks

Study	Population	Sample size and age	VR technology*	Recording device(s)	Stress task used	Study aim	Physiological measures**	Subjective measures***
Annerstedt et al. (2013)	Healthy adults, males	*N* = 30 (27.7, 6.7)	CAVE	ML866 Power Lab	TSST	Assessing stress recovery in virtual forest nature and the relevance of nature sounds	Yes; salivary cortisol, HR, T-wave amp, HRV (HF, LF, LF/HF, TOT)	STAI (short, state subscale)
Biedermann et al. (2024)	Healthy adults	*N* = 39 (25.67, 3.74)	HMD: HTC Vive	Biopac MP150 + Bionomadix module	High-altitude task (elevated plus maze)	Repeated use of the task to study anxiety-related behaviour and the effect on stress of closely duplicating the real environment in VR	Yes; HR, SCL, respiratory rate	STAI (state subscale), 1-item Likert scale
Bolinski et al. (2021)	Healthy adults	*N* = 41 (20.59, 3.07)	HMD: HTC Vive	Empatica E4	Virtual bar or café	Understanding the influence of immersive VR on cognitive restructuring and physiological arousal	Yes; HR, SCL, nsSCRs	PANAS, 1-item Likert scale
Daviaux et al. (2020)	Healthy adults; females	*N* = 12 (28, 9.49)	Driving simulator	Biopac MP36 with EDA	Driving simulation	Simulation of stressful driving events and its potential to be reliably measured by EDA	Yes; ampSCR	VAS
McAllister et al. (2024)	Healthy students	*N* = 53 (21, 3)	HMD: HTC Vive Pro	Polar Verity Sense	Game (active survival)	Examining the impact of caffeine on subjective and physiological stress as well as performance during a virtual shooter game	Yes; HR, salivary cortisol, α-amylase, immunoglobin A	STAI (state subscale)
Morales Téllez et al. (2023)	Healthy adults	*N* = 27 (26, 3.61)	HMD: HTC Vive Pro	Polar H10	Stroop task	Comparison of VR Color-Word Stroop Test and its gamified version to in vivo	Yes; HR	DASS-21 (stress scale)

This table presents a preselection of studies illustrating the variety of the studies included in terms of study aims, the included tasks and the measurement methods. The table with all studies can be found in Appendix Table 5. *HMD = head-mounted display, CAVE = cave automatic virtual environment (stereoscopic projection room), **HR = heart rate, T-wave amp = T-wave amplitude, HRV = heart rate variability, HF = high-frequency power, LF = low-frequency power, LF/HF = low frequency/high-frequency power ratio, SCL = skin conductance level, nsSCRs = non-specific skin conductance responses, ampSCRs = amplitude of skin conductance responses, ***STAI = State-Trait Anxiety Inventory (Spielberger et al., 1983), PANAS = Positive and Negative Affect Schedule (Watson et al., 1988), DASS-21 = Depression Anxiety Stress Scale (Parkitny & McAuley, 2010)

#### Measures

The most commonly used physiological measures were HR (*n* = 65, 59%), skin conductance level (SCL; *n* = 43, 39%), salivary cortisol, and various heart rate variability (HRV)-related parameters (both *n* = 32, 29%). While the physiological recording spanned the entire duration of the experiment, the subjective instruments were most commonly repeated multiple times throughout the session, and the ways of assessing subjective stress levels differed greatly. Among the standardized tools, the most commonly used was the State-Trait Anxiety Inventory (STAI; Spielberger et al., 1983), implemented by 18 studies (16%), followed by the Positive and Negative Affect Schedule (PANAS; Watson et al., 1988) with 7 studies making use of it, and the Subjective Units of Distress Scale (SUDS; Wolpe & Lazarus, 1966) used by 6 studies. It is noteworthy that many authors have used affect- or anxiety-related measures to assess what they referred to as subjective stress. Additionally, nearly a third of studies (*n* = 30, 29%) included a one-item visual analogue scale (VAS; either 0–10 or 0–100) to quantify subjective stress levels, often as part of the baseline and post-stressor assessment.

#### VR technology

With regard to the use of VR technology, a high prevalence of head-mounted displays was observed (*n* = 89, 81%), with a stereoscopic projection screen setup (e.g., the CAVE system) being used in seven experiments. The most frequently used headset was HTC Vive (*n* = 37, 34%), followed by Oculus Rift (*n* = 15, 14%), Sony HMZ-T1 (*n* = 7, 6%), and Oculus Quest (*n* = 6, 5%). In five experiments a driving simulator was used with either a VR head-mounted or a panoramic display. In several instances the VR experience was enhanced through the use of props such as a controller simulating a gun (e.g., Sanz et al., 2015) or tactile-feedback gloves or platforms (e.g., Liu et al., 2019). However, many papers did not report on the VR technology used (*n* = 14, 13%), and even fewer provided details on the development of the VR application itself (*n* = 39, 35%).

### Use of VR for validation of wearables (RQ1)

No studies were identified that utilized VR stress simulations to validate wearables on their convergent validity with a gold-standard reference device. However, several studies implemented wearable devices in a way that hinted at either the construct or convergent validity of such measurement in the context of VR. For example, a study by Schulz et al. (2019) investigating the use of physical props in VR climbing simulations used two electrocardiography (ECG) measurement devices concurrently, a standard ECG recording and a wearable ECG chest strap. The strap was included not with validation in mind, but to provide an additional, less-artifact-prone source of recording during climbing. The visualizations of signals obtained from both devices show comparable data for HR, with slightly higher variability of recording by the chest strap. However, this is an observation based on the visualized graphs, and the concordance of these data was not analysed.

In contrast, Weiß et al. (2020) took an approach indicative of a construct validation effort, as they aimed to assess various signals from different wearable sensors on their alignment with subjective stress reports and their ability to capture stress-related physiological changes at different height exposures. Despite the lack of significant results and a methodological error (lack of baseline recording and potential ceiling effects), the authors note that HR, SCL, and respiration rate consistently increased in response to the stress simulation and that the sensors were sensitive enough to pick up on the subtle changes during the task. This is consistent with other studies (*n* = 12, 11%) which used wearable devices to obtain physiological recording during stressful VR tasks. For example, the four studies that used the Empatica E4 wristband (Bolinski et al., 2021; Macey et al., 2023; Schebella et al., 2020; Zhu et al., 2023) reported significant variations for HR and EDA parameters caused by the stress-inducing tasks or their elements. Collectively, these few existing studies support the use of wearable devices for recording physiological signals during stressful virtual experiences, while at the same time highlighting the limited use of VR for their validation.

### VR stress tasks and their characteristics (RQ2)

To identify the well-established VR stressors, studies were first categorised based on the type of task and the underlying stressors that they targeted. The categories were created by grouping the most prominent tasks on their similarity, with the task having to appear at least three times in independent investigations and be highly aligned in design. For example, Stroop was the only distinct and standardized task that stood out among the cognitive stressors, with the remaining tasks falling under the *Other cognitive tasks* category and including several studies with various math, memory, reaction speed, and navigation elements. Table 3 presents the main emerging task categories for social-evaluative threat, environmental stressors, and cognitive stress, along the tasks’ description and studies that implemented them. The table also includes physiological measures utilized across all the studies with a given task category. While this section summarizes the most prominent tasks identified, lists the variations in the task design, and mentions the noteworthy findings on their stress-inducing elements, the task effectiveness itself is explored in detail later on.

**Table 3:** Emerging task categories classified by type of primary stressor

Type of stressor	Stress test	Task description	Physiological parameters measured across studies	Included in (*N*; pooled sample size; and list of studies)
**Social-evaluative threat** (social cues and judgement as primary source of stress)	**Trier Social Stress Test (VR-TSST)** *Secondary stressor(s): Cognitive,* *Time pressure*	Variations of TSST first developed by Kirschbaum et al. (1993). Most commonly the task uses the following three phases, 5 min each: Participants are given time to prepare (anticipation phase) a short speech to give in front of three judges (speech phase), after which they are asked to perform a sequential, mental subtraction task and report the answer out loud, having to start over if a mistake is made (mental arithmetic phase). This is performed in front of three deliberately unresponsive and formal judges and often contextualized as a high-stakes situation, most commonly a job interview	HR, average RR interval, HRV (SDNN, RMSSD, LF, HF, HF/LF, TOT, DBP, SBP, T-wave amplitude (TWA), RR, EDA (SCL, nsSCRs, nsSCR amplitude, sSCRs), α-amylase, salivary, blood and hair cortisol, epinephrine, norepinephrine [additionally allopregnanolone, platelet TSPO expression and TSPO gene polymorphism rs6971, inflammatory markers (e.g., zonulin)]	**33; 1893**; Annerstedt et al. (2013), Bahr et al. (2021), Delahaye et al. (2015), Domes and Zimmer (2019), Ecker et al. (2024), Fich et al. (2014), García-León et al. (2019), Halbeisen et al. (2023), Heming et al. (2024), Jönsson et al. (2010), Jönsson et al. (2015), Kelly et al. (2007), Kothgassner, Hlavacs, et al. 2016)), Kothgassner et al. (2021), Laskowitz and Huffstadt (2023), Lee et al. (2023), Linninge et al. (2018), Liszio and Masuch (2019), Liu and Zhang (2020), Macey et al. (2023), Montero-López et al. (2016), Montero-López et al. (2018), Mostajeran et al. (2020), Santos-Ruiz et al. (2012), Schebella et al. (2020), Schote et al. (2022), Schröder and Mühlberger (2023), Shiban et al. (2016), Vatheuer et al. (2021), Verdejo-Garcia et al. (2015), Weber et al. (2024), Zimmer, Buttlar, et al. (2019a, 2019b), Zimmer, Wu, et al. (2019)
	**Virtual bar/café (or other similar social setting)** *Secondary stressor(s): Environmental*	In this type of stressor participants are exposed to a social environment, in which several social cues can be manipulated to induce stress. These most commonly include *crowdedness*, *ethnicity* of the avatars (similar or opposite to that of participant) and their *hostility*. The environment is usually a café or a bar, which participants get to explore while performing an engaging yet simple task (e.g., identifying avatars with numbers on them)	HR, SCL, nsSCRs [additionally strong focus on behavioural (e.g., interpersonal distance) and subjective measures, especially subjective units of distress]	**8; 870;** Bolinski et al. (2021), Counotte et al. (2018), Geraets et al. (2018), Hartanto et al. (2014), Isnanda et al. (2014), Pot-Kolder et al. (2018), Veling, Counotte, et al. (2016a, 2016b), Veling, Pot-Kolder, et al. (2016a, 2016b)
	**Other social-evaluative tasks** *Secondary stressor(s): Cognitive*	This category covered other speech/presentation tasks next to interaction with aggressive or judgemental virtual avatars, as well as social exclusion. While for some studies this interaction was one-on-one, in most cases the social evaluation was elicited by an audience or a group of people	HR, HRV (RMSSD, HF, LF/HF), respiratory sinus arrhythmia (RSA), RR, EDA (SCL, sSCRs), BP, root mean square EMG, salivary cortisol, epinephrine, norepinephrine, testosterone	**9; 252;** Ban et al. (2024), Bosse et al. (2018), Giron et al. (2023), Kotlyar et al. (2008), Kothgassner, Felnhofer, et al. (2016a, 2016b), Kothgassner, Hlavacs, et al. (2016), Pallavicini et al. (2013), Schudy et al. (2023), Van Dammen et al. (2021)
**Environmental** (elements of environment and the perceived physical relation to these as source of stress)	**High-altitude task** *Secondary stressor(s): Phobia/fear, Physical*	This category of tasks includes stressors designed to induce fear through exposure to heights. Participants are most commonly subjected to heights by moving elevators or platforms, planks and beams to walk on, or, less commonly, cliffs. The task to be performed is often walking to the edge of the structure utilized and is sometimes combined with additional movement or cognitive task elements. Often physical props outside VR are used and closely aligned with their virtual versions, e.g., a real physical plank	HR, average RR interval, HRV (RMSSD, SDNN, HF, LF, LF/HF), EDA (SCL, nsSCRs), RR, α-amylase, salivary cortisol	**17; 803;** Biedermann et al. (2024), Diemer et al. (2016), Dietz et al. (2022), Finseth et al. (2018), Liu et al. (2019), Lugrin et al. (2016), Martens et al. (2019), Meehan et al. (2003), Newman et al. (2020), Nouri et al. (2022), Phillips et al. (2010), Phillips et al. (2012), Schulz et al. (2019), Seinfeld et al. (2015), Skarbez et al. (2018), Weiß et al. (2020), Zhu et al. (2023)
	**Emergency simulation (active)** *Secondary stressor(s): Physical,* *Time pressure*	This type of task constitutes various emergency scenarios, such as explosions, fires, floods or accidents resulting in the need to actively escape, minimize damage, or rescue injured avatars. These actions are often purposefully ineffective to increase stress through the high uncontrollability and unpredictability of the situation. Most of these tasks take place in confined spaces, mostly underground (e.g., parking lots or subway stations), and with multiple multisensory cues, such as flickering lights, sound alarms and even actual smoke	HR, HRV (HF), EDA (SCL), BP, pupil dilation	**7; 312;** Breuninger et al. (2017), Dong et al. (2020), Hirt et al. (2020), Lin et al. (2020), Schweizer et al. (2017), Schweizer et al. (2019), Weiß et al. (2022)
	**Trauma/horror scenario (passive)** *Secondary stressor(s): Emotional, Physical*	In this category of tasks, stress is induced through passive exposure to traumatic or horror virtual content. Participants experience, through a first-person perspective, events such as car accidents with traumatic aftermath, abandoned horror-like environments with flickering lights, distressing sounds and sudden frightening stimuli	HR, EDA (SCL, nsSCRs, SCRs amplitude), skin temperature, EEG (beta), facial EMG	**5; 183;** Baldini et al. (2022), Baptie et al. (2021), Hanshans et al. (2024), Heffer et al. (2023), Park et al. (2020)
	**Game (active survival)** *Secondary stressor(s): Physical;* *Auditory; Cognitive*	This type of stressor includes strong gamification elements, where participants are actively interacting with the environment under a dynamic, high-stakes game scenario. Tasks in this category require a high level of engagement, most commonly while fighting off enemies or monsters by shooting, often in dark, low-light settings with unsettling sounds and limited resources. Additionally, task penalties are often part of this gamification, e.g., depleting oxygen or health levels	HR, average RR interval, HRV (RMSSD, SDNN, HF, LF, Poincaré plot-derived parameters) EDA (SCL, nsSCRs), α-amylase, salivary cortisol, BP, EEG parameters for different frequency bands, Immunoglobulin A [additionally strong focus on behavioural data; motion from VR headset, button presses, speech analysis]	**10; 264;** Brambilla, Boccignone, Borghese, Croci, et al. (2023a), Brambilla, Boccignone, Borghese, Chitti, et al. (2023b), Legkov et al. (2017), McAllister et al. (2024), Misztal et al. (2020), Pratiher et al. (2022), Sahoo et al. (2021), Sanz et al. (2015), Spangler et al. (2021), Tao et al. (2022)
	**(Un)controlled motion** *Secondary stressor(s): Phobia/fear; Physical*	This task category includes stressors strongly related to movement of the environment while the participant remains static resulting in a heightened physiological reaction due to the visuo-vestibular dissociation. Although height is often present as a stress-inducing element, this task was categorized separately due to its prominent motion-sickness-inducing nature. The most common use of this stressor is a roller coaster ride experience with sudden changes in speed, direction and elevation	HR, average RR interval, HRV (SDNN, distance Poincaré index, LF, HF, LF/HF), EDA (skin resistance, not SCL)	**4; 36;** Bu (2020), Lebamovski and Gospodinova (2023), Ostermeier and Schmoll (2022), Sabo et al. (2018)
	**Driving simulation** *Secondary stressor(s): Physical; Auditory; Cognitive*	This task requires engagement in driving through the use of driving simulators and under dynamic, unpredictable conditions. The stress-inducing elements are most commonly environmental, such as bad visibility and weather, highly increased traffic, aggressive or unpredictable behaviour of other road users, accompanied by strong auditory cues (e.g., honking). Additionally, some versions gamify the experience through addition of racing	HR, HRV (RMSSD, HF), EDA (SCL, nsSCRs, event-based ampSCRs), BP, cardiac output, total peripheral resistance	**5; 808** Agrawal and Vemuri (2019), Daviaux et al. (2020), Eudave and Valencia (2017), Turner et al. (1997), Wang et al. (2009)
**Cognitive** (cognitive demands as main source of stress)	**Stroop** *Secondary stressor(s):* *Social-evaluative threat; emotional*	This category spans various virtual adaptations of the Color-Word Stroop Test (Stroop, 1935), often including both the congruent and incongruent trials and taking place in an office or a neutral setting. Often an emotional component is added through negative-valanced words or the presence of judgemental avatars present to increase social-evaluative threat	HR, HRV (RMSSD, index by a device algorithm), EDA (SCL, nsSCRs), EEG (parameters for different frequency bands)	**8; 193;** Donahue and Shrestha (2019), Gradl et al. (2019), Kamińska et al. (2021), Kerous et al. (2020a), Kerous et al. (2020b), Mevlevioğlu et al. (2022), Morales Téllez et al. (2023), Poguntke et al. (2019)
	**Other cognitive tasks** *Secondary stressor(s): Time-pressure; auditory*	This broad category spans a wide range of tasks where cognitive demands form the biggest stress-inducing element. These cognitive demands are produced by math, memory, or navigation tasks, or a combination of those. These tasks are often performed under time pressure, with auditory disturbances or environmental stressors and sometimes with social evaluation of performance	HR, HRV (RMSSD, SDNN, HF, LF, LF/HF, triangular index, heart deceleration capacity, pNN20 and pNN50, approximate and sample entropy of NN intervals, Poincaré plot-derived parameters), EDA (SCL, nsSCRs, amplitude of nsSCRs), RR, salivary cortisol, skin temperature	**8; 496;** Bullinger et al. (2005), Cho et al. (2017), Eftekharifar et al. (2021), Yin et al. (2020), Kristensen et al. (2020), Legkov et al. (2017), Rodrigues et al. (2020), Rodrigues et al. (2021)

#### Most prominent stress tasks

##### Trier Social Stress Test as the most common stressor for social-evaluative threat

As can be seen in Table 3, nearly half of all studies used social-evaluative threat as a primary stressor, with the VR version of TSST being the most common task identified overall, spanning the highest pooled sample and the most physiological measures used across all studies. While most authors replicated the original TSST quite closely, some introduced slight variations, for example, two judges instead of the regular three (Kothgassner, Hlavacs, et al., 2016) and shortening the preparation phase to 3 min (Liu & Zhang, 2020; Schote et al., 2022; Schröder & Mühlberger, 2023; Zimmer, Buttlar, et al., 2019a, 2019b) or the mental arithmetic phase to 1–2 min (Laskowitz & Huffstadt, 2023), with Lee et al. (2023) even removing the preparation phase completely. Several authors have further adapted the TSST by replacing the three judges present in the original version with a large audience, for example, 80 avatars in a lecture hall setting (Delahaye et al., 2015; Santos-Ruiz et al., 2012), with the assumption that a larger number of virtual assessors will increase the stress response through the higher level of social-evaluative threat. However, Mostajeran et al. (2020) systematically manipulated the size of the virtual audience (3, 6, or 15 avatars) and found that it was the condition with three judges that produced the highest heart rate reactivity. Similarly, one study created an exact VR replica of the lab in which the experiment took place (Shiban et al., 2016), yet Zimmer, Wu, et al., 2019), who manipulated this TSST task element (exact lab replica vs a novel virtual space), found no differences in either subjective or physiological stress levels. Another noteworthy exploration is the height manipulation of the interviewee in relation to the judges (Macey et al., 2023), where the participant either stands looking down at the assessors seated in front of them (with the height difference artificially increased) or sits down in front of the judges’ panel. The findings suggest that the seated, low-point-of-view condition is more stress-inducing as reflected in the higher rate of non-specific skin conductance responses (nsSCRs) and subjective stress reports. Despite all these adaptations, the TSST was still the most unified in its procedure and design relative to the stressors described below.

##### Perceived height in a virtual environment as source of stress

High-altitude tasks have emerged as the most commonly used among the surprisingly rich group of environmental stressors. It is noteworthy that this task, as well as many others classified as environmental, includes a strong physical component through which the participant is subjected to or required to take action based on the dynamic elements of a virtual environment. In this specific case, it is the increase in perceived height that is the main source of stress. However, the height to which participants are subjected seems to vary between studies, with no standards on the optimal levels. Several studies evaluated the changes in height in a systematic way and provide support for higher physiological reactivity at increased heights (Dietz et al., 2022; Seinfeld et al., 2015; Weis et al., 2020); Zhu et al., 2023). The study by Zhu et al. (2023) seems to be the most comprehensive manipulation and additionally combines it with varied elevation of an actual physical plank. Their findings suggest that the most extreme virtual height together with real physical elevation is the most stress-inducing combination for both physiological and subjective stress. However, elevating the physical plank seems to be most effective at low virtual height, and its effect on stress is disproportionately low at the extreme virtual height. It is instead the more extreme elevation (virtual or physical) that will serve as the most significant perceived threat. However, it must be noted here that this study did not include a condition in which the extreme VR height would be combined with no physical plank present. It hence remains unknown to what degree the inclusion of the physical plank at extreme virtual heights is conducive to the stress response, an essential consideration since such a physical plank or structure was present in all of the studies identified. Moreover, the lack of added value of replicating the experiment’s environment in VR previously mentioned for TSST was also observed for this task, with no differences found between the exact replica of the lab (with floor tiles falling to reveal a pit) and a novel, abstract VR space (Biedermann et al., 2024; Phillips et al., 2010). Among other elements which influence the stress response is the subject’s gaze direction, with larger stress reactivity (HR, SCL) when looking down than looking straight ahead (Diemer et al., 2016), and elevator music as a facilitator of post-stressor recovery (Seinfeld et al., 2015). Lastly, the task seems effective regardless of the reported level of fear of heights, including the lack thereof (Finseth et al., 2018).

##### Virtual Stroop Color and Word Test as a cognitive stressor

Similarly to TSST, the prominence of the Stroop Color and Word Test (Stroop, 1935) as an established cognitive stressor translates to its common use in VR. Authors often referred to the task’s ability to not only induce stress but to simultaneously assess cognitive performance as the added value inherent in its design. Interestingly, most of the studies used VR to introduce additional distressing elements to the regular VR-Stroop, supplementing it with gamification (Morales Téllez et al., 2023), social-evaluative threat (Kerous et al., 2020a, 2020b), or spatial stimuli (the Stroop Room; Gradl et al., 2019) or replicating the Emotional Stroop task with negatively valanced words (Mevlevioğlu et al., 2022). For example, Morales Téllez et al. (2023) compared a regular VR-Stroop with its gamified version in which the participant was placed at a football pitch and needed to throw a virtual ball to select the correct answer. They found a greater increase in HR and higher immersion for the gamified condition, while also showing that it was perceived as less stressful and even as enjoyable. However, the authors do not reflect on the physical nature of the gamified task and its likeliness to influence HR. Similarly, an addition of social evaluation through judgemental avatars showed higher cardiovascular and especially electrodermal reactivity when compared with the regular VR-Stroop condition (Kerous et al., 2020b) or with a social-conflict scene with mockery by the judges (Kerous et al., 2020a). Additionally, the authors claim that EDA was more reflective of non-interactive social simulation, with cardiovascular measures more indicative of task-related stress evoked by Stroop. Interestingly, Gradl et al. (2019) translated the Stroop task into a VR Stroop Room, where a correctly coloured wall needs to be selected. While they found no subjective stress reactivity, they reported the task to be effective for multiple cardiovascular and especially EDA parameters (e.g., average increase in HR of 19% while SCL increased by 63% on average and nsSCRs by 135%). Lastly, while the Emotional Stroop was successfully recreated in VR (Mevlevioğlu et al., 2022), the study does not provide insights on task effectiveness for either physiological or subjective stress induction.

### Effectiveness of VR stress induction tasks and their measurement (RQ3)

**Fig. 2:**
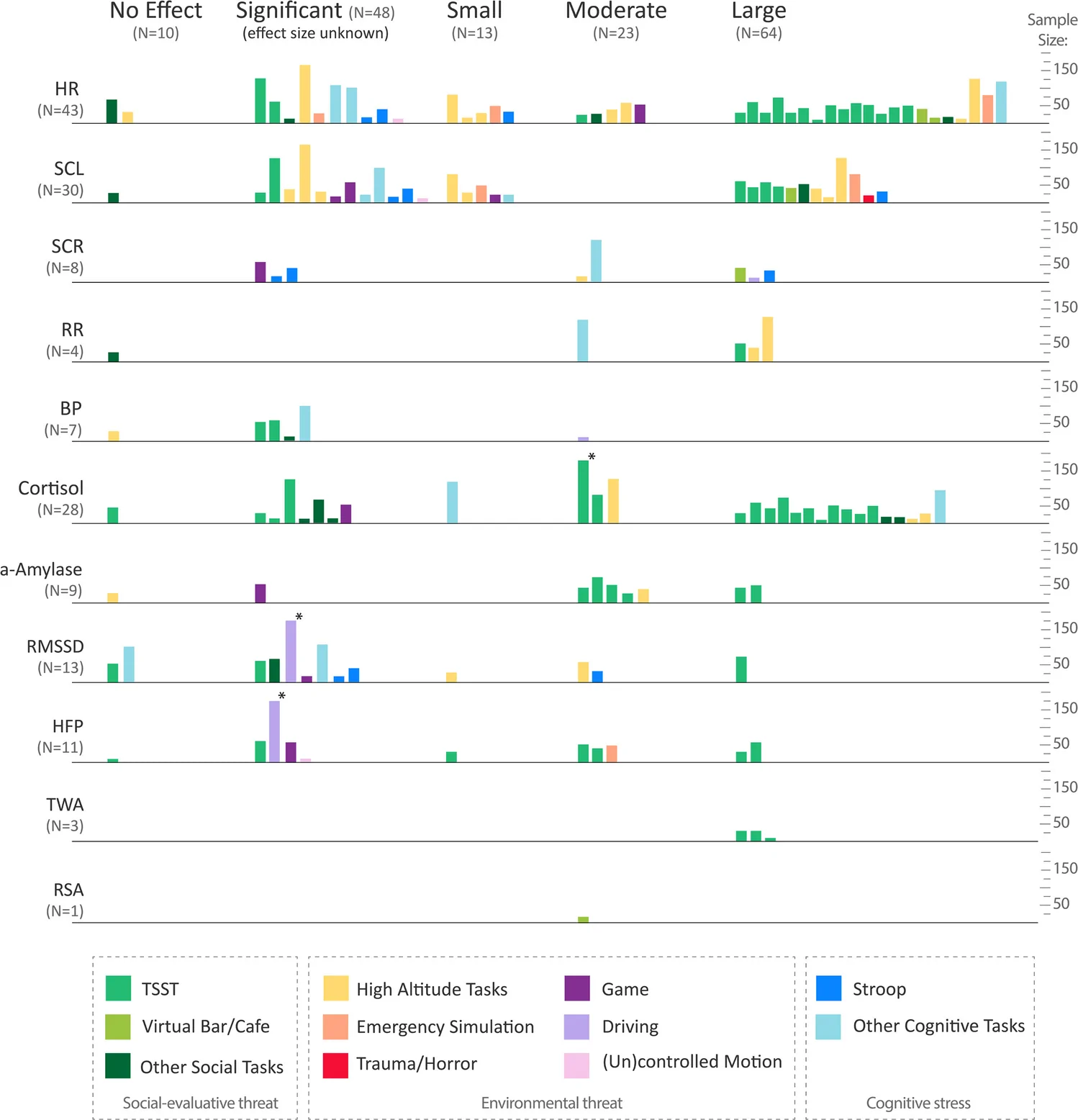
Evidence on task effectiveness for the preselected physiological parameters. *Note.* Each bar represents a study which reported relevant results on a particular parameter. The height of the bar indicates sample size. Colour of the bars represents the task. The three most prominent tasks in each stressor category are TSST (vivid green) for social stressors, high-altitude task (yellow) among environmental stressors, and Stroop (bright blue) as a most common cognitive stressor. *The sample size of these two studies is larger than visualized (TSST, *N* = 274; Driving, *N* = 735), but for the purpose of this graph the height of the bar was kept smaller

The results for each study were synthesized with regard to the effectiveness of the task used, and this detailed overview (along with all other extracted information) can be found in Supplementary 2. In line with the primary goal of this review, the most prominent VR stress induction tasks were then further evaluated for their reported effectiveness. Figure 2 presents a harvest plot of all the available results on physiological parameters of interest and for all the tasks, including all the reported nonsignificant and significant changes where no effect sizes were specified, which, as visible in the plot, was often the case. The plot highlights the dominance of social stressors, especially the TSST (bright green), with most studies showing moderate to large effects, though it also entails a strong cognitive component. Evidence for purely cognitive stressors, by contrast, is limited. What also becomes clear is the variance in sample size and the relatively small sample size of some of the studies, with over half the samples smaller than 50, and 28 of those with only 30 or fewer participants. While the high-altitude task (yellow) is also supported by various studies and outcome measures, it can be seen that evidence for Stroop (bright blue) is still lacking. Furthermore, it is apparent in the plot that HR, SCL, and (salivary) cortisol emerge as the most commonly reported measures of stress with consistent and robust findings, while measures such as respiratory sinus arrhythmia (RSA) and T-wave amplitude (TWA) appear underutilized. Although tasks differ in their effectiveness, all of the stressors included consistently resulted in a measurable physiological stress response of a varying degree, with very few reports of no effects and with several studies with large sample sizes (*N* > 100) adding to the reliability of the significant results.

#### Effectiveness of VR-TSST

The VR-TSST emerges not only as the most widely explored task but also as a consistently effective paradigm, with moderate to (predominantly) large effect sizes for HR, SCL, α-amylase, and cortisol, although one study (Shiban et al., 2016) reported no effect of VR-TSST on salivary cortisol. The effectiveness of TSST is also most varied for the HRV high-frequency (HF) component, with inconclusive findings and with Jönsson et al. (2010) reporting none and Annerstedt et al. (2013) only small effects for this parameter. Similarly, one investigation found no effects of VR-TSST on the root mean square of successive differences (RMSSD; Heming et al., 2024). However, the generally stable nature of TSST’s effectiveness is confirmed by the large effect sizes of the remaining measures. The physiological effectiveness is also aligned with the subjective reports, with all the studies that measured it (N = 21) reporting significant and (when provided) moderate to large increases.

##### Comparison with in vivo TSST

Furthermore, the evidence for VR-TSST’s effectiveness is strengthened by the seven studies which compared it with in vivo TSST. The in vivo condition was most commonly the traditional lab version of the task (Ecker et al., 2024; Kelly et al., 2007; Kothgassner et al., 2021; Mostajeran et al., 2020; Shiban et al., 2016; Zimmer, Buttlar, et al., 2019a, 2019b), with one study utilizing a screen projection (Montero-López et al., 2016). Among all the investigations, VR-TSST consistently induced significant physiological stress responses, successfully activating both the hypothalamic–pituitary–adrenal (HPA) axis and ANS and clearly replicating the parameter-specific temporal response profiles typical of TSST (e.g., the peak HR usually happening during speech). However, the effect sizes of these responses are usually smaller when evoked by VR-TSST. For example, Kothgassner et al. (2021) showed that the VR-TSST resulted in an average 70% increase in salivary cortisol from baseline while the in vivo condition increased cortisol by 90%. An even larger difference was observed by Kelly et al. (2007) with a 30% increase for the VR-TSST versus 90% for the traditional TSST. The less pronounced cortisol response to the VR version of this task is further confirmed by three other studies (Ecker et al., 2024; Mostajeran et al., 2020; Zimmer, Buttlar, et al., 2019a, 2019b). However, the superiority of the in vivo TSST for other parameters is not univocally supported. While Mostajeran et al. (2020) and Zimmer, Buttlar, et al. (2019a, 2019b) found a similar pattern of significant but weaker HR responses relative to the in vivo condition, other authors report no differences for either HR or RMSSD (Ecker et al., 2024; Kothgassner et al., 2021; Shiban et al., 2016). Similarly, the elicited response does not seem to differ for SCL (Mostajeran et al., 2020; Shiban et al., 2016; Zimmer, Buttlar, et al., 2019a, 2019b), with one study notably reporting a stronger SCL increase in VR-TSST than in the in vivo display condition (Montero-López et al., 2016). The lack of difference was also visible in the subjective ratings, with none of the studies reporting a significantly higher subjective stress in vivo, and with Shiban et al. (2016) finding a larger subjective stress response in VR than in vivo, with VR-TSST considered more challenging. Their findings additionally hint at a dissociation of physiological and subjective responses to the task, as the in vivo condition resulted in higher cortisol levels while VR-TSST was more stressful subjectively. Despite these differences and the apparent attenuation of physiological reactivity, all the authors conclude that the VR-TSST is an effective paradigm for multimodal stress induction, which is consistent with the general effectiveness of the task described above.

#### Effectiveness of the high-altitude task

In comparison with TSST, the high-altitude task is effective in inducing a physiological response, but it is less investigated and the effect sizes vary more. While cortisol and respiration rate remain parameters with moderate to large effects, results seem inconclusive for heart rate, with small to large effects being observed. In contrast to TSST, the effect sizes of EDA measures, RMSSD, and α-amylase decrease to small and moderate, with one report of no increase in blood pressure (Martens et al., 2019). Similarly, the subjective stress findings support the effectiveness of the task in general (Biedermann et al., 2024; Diemer et al., 2016; Martens et al., 2019), but some inconsistencies were observed, with Liu et al. (2019) reporting large affect effects while Meehan et al. (2003) found nonsignificant results in a sample of 164 participants despite the slight increase in self-reported values and significantly increased HR and SCL. Interestingly, it appears that the design of the task has an influence on its effectiveness. Namely, the studies including an open platform or “pit” design, with the floor elements disappearing to reveal a considerable drop, showed no effect on blood pressure (BP) or on α-amylase, no-to-small effects on HR, and only small effect sizes for SCL and RMSSD (Martens et al., 2019; Meehan et al., 2003; Phillips et al., 2010). Conversely, the tasks utilizing a narrow virtual plank-like structure at high altitude often produced large effect sizes for HR and salivary cortisol (Dietz et al., 2022; Finseth et al., 2018; Liu et al., 2019).

##### Comparison with in vivo height exposure

In contrast to TSST, only one study was identified that evaluated the difference between VR and in vivo height exposure. This high-altitude climbing task has been shown to induce a stress response comparable to that of the real-life in vivo climbing condition, with comparable EDA and HR increases from the rest baseline (Schulz et al., 2019). Notably, the VR condition resulted in higher subjective stress ratings, a finding previously observed for VR-TSST. Overall, although the task seems to be slightly less effective than VR-TSST in eliciting large physiological responses, it is still an effective multimodal stressor. It is also particularly VR-suited and not easily reproducible in vivo under laboratory conditions, as illustrated by the lack of comparisons.

### Effectiveness of VR-Stroop

While some indication of VR-Stroop effectiveness for physiological stress reactivity exits, it remains limited, and the effect sizes of the responses remain unknown, with only two connected studies (out of eight) by Kerous et al. (2020a, 2020b) reporting results in a way that shows significant changes in HR, SCL, SCRs, and RMSSD in response to the task itself. Interestingly, in both these studies, the authors used Stroop as a replacement for the TSST’s cognitive math task with the aim of increasing consistency between participants’ task-related stress and lowering habituation effects. This also entails however that the performance was socially evaluated. In turn, the VR Stroop Room (Gradl et al., 2019), the spatial virtual recreation of the task’s elements, appears to be the most promising VR version explored so far, with small to medium effects for HR and RMSSD and large effect sizes for SCL and SCRs. However, the general effectiveness of VR-Stroop for stress induction is further brought into question by the inconsistent subjective results. While Kerous et al. (2020b) did find increased levels of subjective stress, four other studies did not (Gradl et al., 2019; Kamińska et al., 2021; Morales Téllez et al., 2023; Poguntke et al., 2019), with participants describing a state of enjoyment or focus rather than stress.

#### Comparison with in vivo Stroop

On the other hand, evidence for the utility of VR-Stroop can be drawn from several in vivo comparisons showing comparable physiological reactivity. Morales Téllez et al. (2023) evaluated the VR-Stroop, its gamified VR version, and the in vivo test, with all conditions resulting in increased HR but with VR tasks achieving higher HR increases, especially for the gamified condition. This trend was confirmed by Poguntke et al. (2019), who evaluated an in vivo Stroop, a VR one, and a VR version with uncontrolled movement (rotation) of the environment. Here again, all conditions resulted in comparable effects for HR and HRV (unspecified in terms of parameters), with no significant differences but with the VR conditions resulting in slightly heightened physiological responses. Despite such results, and due to the lack of subjective stress experienced in the regular VR condition, the authors conclude that the task in itself is not sufficient as an effective VR stressor and requires additional distressing elements, such as uncontrolled movement, as that was subjectively seen as more stressful. However, Donahue and Shrestha (2019) showed comparable effects of in vivo and VR-Stroop for both performance and subjective distress, unfortunately not utilizing any physiological measures. In general, the scarce evidence suggests that physiological and performance-based outcomes are comparable between in vivo and VR-Stroop tasks, with inconsistencies observed on the subjective level, and with surprisingly no exploration of effect sizes of physiological stress responses.

#### Motivations for selecting a VR stress task (RQ4)

Among all studies, the most common reason identified for choosing a specific stress task was purely pragmatic and refers to a task’s proven ability to induce a physiological stress reaction. This is especially visible for TSST, with many authors citing the existing body of evidence on its stress-inducing effects and the validated VR adaptations (e.g., Annerstedt et al., 2013; Bahr et al., 2021; Delahaye et al., 2015; García-León et al., 2019; Zimmer, Buttlar, et al., 2019a, 2019b). Even if physiological responses were recognized as attenuated in VR versions versus in vivo, many authors listed additional pragmatic considerations to substantiate creating VR stress paradigms. Here, the reasoning was often not task-specific and instead transitioned towards the added value of VR for stress induction in general. Next to high standardization and replicability, VR was seen as a way of reducing personnel requirements and burden, especially in the context of social-evaluative threat (e.g., Counotte et al., 2018; Kelly et al., 2007; Schebella et al., 2020; Van Dammen et al., 2021). VR was additionally perceived as a means of creating engaging but controlled environments which are either less immersive in other media or difficult to control in vivo (e.g., Mostajeran et al., 2020; Schudy et al., 2023; Schweizer et al., 2019; Tao et al., 2022). This was especially true for the tasks including a large setting and number of avatars, such as bars, cafés, or auditoriums with audiences (e.g., Pallavicini et al., 2013; Veling, Counotte, et al., 2016a, 2016b). Safety and ethical concerns were frequently raised regarding the social stressors, and the same reasoning applied to the environmental tasks (e.g., high-altitude, driving, or emergency simulations; Pot-Kolder et al., 2018; Weiß et al., 2022; Zhu et al., 2023). What was also commonly observed was a task being chosen simply to match the aims of the study. For example, for the cognitive stressors, Stroop authors often referred to their ability to not only induce stress but to simultaneously assess cognitive performance (e.g., Donahue & Shrestha, 2019). Similarly, the virtual bar/café category allowed researchers to manipulate relevant social stressors to better understand paranoid ideation in psychosis (Pot-Kolder et al., 2018). All this, and the flexibility of stress induction in VR combined with easy scalability (for example, combining multiple stressor elements), paints VR as an efficient medium for controlled stress induction while enabling the creation of tasks otherwise challenging in regular lab settings.

In comparison, detailed theoretically grounded motivations were less common and rarely moved beyond short descriptions of the already proven ability of the task to evoke a physiological stress reaction. The link to theory was again predominantly present for TSST studies, where the task was often reported to reliably activate the HPA axis and the autonomic nervous system due to the collection of stress-inducing elements, mostly social-evaluative threat and uncontrollability (e.g., Jönsson et al., 2015). Overall, less than a third of investigations mentioned elements of existing stress theories or included established psychophysiological frameworks. Beyond the HPA and ANS stress response models, concepts such as allostatic load, toughness theory, or attention restoration theory were used to anchor the stress-inducing properties of a task (e.g., Heming et al., 2024; Park et al., 2020). The most comprehensive theoretical approach seems to be taken by the developers of the Immersive Multimodal Virtual Environment Stress Test (IMVEST; Rodrigues et al., 2021), where the design of the tasks was heavily guided by theoretical guidelines on acute stress elicitation by Dickerson and Kemeny (2004). In their scenario, performance on a mental-arithmetic task carries negative effects based on too slow or incorrect responses, with tiles on the floor exploding randomly to reveal a virtual height. The addition of social evaluation in the form of negative feedback strengthens the stress-inducing task properties. The task combines cognitive, environmental, and social-evaluative elements, and the difficulty level is additionally adaptive throughout the test based on the subject’s performance. IMVEST was found effective in eliciting a stress response, as reflected in a large effect size for increased HR, moderate effects for respiration rate and nsSCRs, and a small but significant increase in salivary cortisol. Another prominent example of purely theoretical motivation for creating a given VR stressor was the collection of high-altitude tasks utilizing the elevated plus maze paradigm. Here, the stress–anxiety animal behavioural models such as approach–avoidance conflict formed the theoretical base for creating a matching VR task in order to study human anxiety-related behaviour (e.g., Biedermann et al., 2024).

Some studies mentioned increased ecological validity as a motivation for creating VR stressors which are more subtle but more reflective of daily life stress—for example, a customer-service simulation (Ban et al., 2024) or driving scenarios (Daviaux et al., 2020). We also observed that a task was sometimes used to extend the existing theories, such as employing trauma analogues to extend models of post-traumatic stress (Heffer et al., 2023). In general, practical and theoretical motivations were often intertwined, with the collection of pragmatic considerations, evidence-based support for task effectiveness, and, to a lesser extent, theory-backed positioning of the stressor elements in relation to stress reactivity.

### Availability of VR stress induction tasks (RQ5)

While previous successful use of a task in VR was often mentioned as a reason for including it in a study, the availability of VR stress tasks as open-source software is surprisingly limited. For the majority of paradigms, the development and availability of the task is not sufficiently described. Even though studies often mentioned open-source software used to create or run the simulation—for example, Unity3D or Steam—little detail was provided on the task simulation itself. Only 37% of studies transparently reported either their custom development of the application, such as with the help of their university’s research lab or with commercial partners, or the use of existing commercial solutions. We identified only two examples of open-access VR stress test applications. The VR Stroop Room (Gradl et al., 2019) appears to be entirely available for download on the StroopRoom GitHub repository (https://github.com/mad-lab-fau/StroopRoom). Additionally, the aforementioned IMVEST project (Rodrigues et al., 2021) is accessible online (see original paper) upon request. Next to those, NeuroVR 2.0 (Riva et al., 2011) was identified as open-source software for VR stress simulations, but it appears to no longer be supported.

Among more recent publications, several commercial solutions stood out. The most frequently mentioned industry partner, CyberSession (VTplus GmbH, Würzburg, Germany), was present in eight studies, with most using it for TSST (Bahr et al., 2021; Halbeisen et al., 2023; Schote et al., 2022; Shiban et al., 2016; Vatheuer et al., 2021; Zimmer, Buttlar, et al., 2019a, 2019b; Zimmer, Wu, et al., 2019) and one to simulate the high-altitude task (Diemer et al., 2016). Another prominent example of a commercial partner was CleVR (Delft, the Netherlands), providing a virtual bar/café simulation to some of the studies utilizing this task (Counotte et al., 2018; Geraets et al., 2018; Veling, Counotte, et al., 2016a, 2016b; Veling, Pot-Kolder, et al., 2016a, 2016b). In turn, it appears that CleVR used Vizard software (WorldViz, Santa Barbara, CA, USA) to create their applications, together with one other study, again for TSST (Montero-López et al., 2016). Next to those, *Lunchroom Zondag* (IJsfontein, Amsterdam, Netherlands; Bolinski et al., 2021) and *Virtually Better* (Virtually Better, Inc., Atlanta, GA, USA; Kelly et al., 2007; Kotlyar et al., 2008) were identified. All of these appear to be commercial solutions offering both pre-made and custom-made applications, but they are not freely distributed.

Furthermore, some authors have used existing VR simulations available via Steam, a platform where multiple VR applications can be purchased (Valve Corporation, 2003). These include the *City Car Driving* application (Eudave & Valencia, 2017), *Epic Roller Coasters* (Ostermeier & Schmoll, 2022), and the *NoLimits Roller Coaster Simulation* (Sabo et al., 2018). These applications are not open-source but can be purchased relatively cheaply, allowing for standardization of tasks across studies. However, these stand-alone applications are targeted at consumers and give little room for research-relevant adjustments, such as controlling the flow of the experiment or adding event markers. Such experimental control is in turn increased with the custom-made commercial solutions presented earlier.

## Discussion

This scoping review provides support for the use of VR for stress induction. This is evidenced by consistent elicitation of autonomic, endocrine, and subjective stress responses across a wide range of tasks and context, and with HR, EDA, and HRV emerging as the most frequent and responsive autonomic markers. Among the identified VR stress paradigms, VR-TSST showed the strongest and most consistent multimodal reactivity, followed by the high-altitude task. Other environmental and cognitive tasks are less represented and produce more varied and often smaller but still reliable responses. Despite this demonstrated effectiveness of VR stress induction, we found that wearables are rarely used as primary physiological measurement devices in VR studies, and no study has yet used VR stress induction to validate wearable signals against a gold-standard reference. However, the several studies using wearables in VR setups (Bolinski et al., 2021; Hirt et al., 2020; Kristensen et al., 2020; Macey et al., 2023; Morales Téllez et al., 2023; Newman et al., 2020; Schebella et al., 2020; Schulz et al., 2019; Tao et al., 2022; Weiß et al., 2020; Zhu et al., 2023) suggest the feasibility of such recording and its potential to capture stress-related physiological changes. Furthermore, task selection across studies was often substantiated through pragmatic considerations rather than by theoretical grounding. Lastly, despite a large number of tasks identified, openly available VR stress tests suitable for research remain scarce, adding to the heterogeneity across studies utilizing the same tasks with, sometimes substantially, different designs. Together, these findings reveal that VR stressors are diverse yet effective, that the most commonly used autonomic markers align well with what current wearables can measure reliably (e.g., HR), and that the field is ready for, but has not yet taken, the next step of using VR as a controlled, ecologically relevant environment for systematic wearable validation. In the following sections, we interpret these findings in light of (1) what was already known about the effectiveness of VR stress induction tasks, (2) the potential of VR for wearable validation, and (3) the need for more theoretically grounded selection of stress paradigms, as well as (4) for harmonization and higher transparency in reporting on VR development and task availability.

### VR as a tool for stress induction

The results of this review support the use of VR for stress induction by showcasing the wide scope of tasks available, common variations in their design, and evidence for task effectiveness. While the existing literature on stress induction in VR has so far focused either on TSST in separation (Helminen et al., 2019, 2021) or on TSST as superior to all other stressors (Van Dammen et al., 2022), this review aimed to merge the fragmented evidence and present a more comprehensive, bottom-up synthesis of the VR stress induction field. By listing the large number of stress tests currently employed and with additional mapping of each task onto the underlying stressors and the evidence on task effectiveness, we hope to contribute to more informed task selection for future studies. It is not surprising that VR-TSST has emerged as the most common and most effective paradigm, confirming its leading position for broadly applicable stress elicitation. This is in line with current meta-analytic literature positioning TSST as a highly effective stress task for HPA and ANS reactivity both in the laboratory (Dickerson & Kemeny, 2004; Helminen et al., 2021) and in VR (Helminen et al., 2019, 2021; Van Dammen et al., 2022). Although the (VR-)TSST seems to dominate the field of stress induction, the environmental stressors category was surprisingly abundant. The high-altitude task, consistently producing significant increases in HR and SCL, appears to be a valid, non-social stressor, additionally utilizing the full potential of VR to create an immersive sense of physical danger while ensuring participants’ safety.

In general, all the tasks identified as unique task categories were shown to be effective across multiple physiological parameters with consistent significant changes, despite less evidence overall for tasks other than TSST and the high-altitude task. In fact, the few reports of no effects being found belong mostly to studies utilizing TSST and high-altitude tasks. It is noteworthy that these studies have rather small sample sizes and that the overall number of studies for those tasks is higher, naturally producing a more varied picture. For example, for VR-TSST it could be that cortisol and HRV parameters are less reliable or less reactive than other measures. This would be in line with our and previous findings that VR-TSST appears to produce smaller cortisol responses than its in vivo counterpart, while ANS parameters remain attenuated but more often comparable. Similarly, Van Dammen et al. (2022) found no effects of VR-TSST for RMSSD and high-frequency/low-frequency (HF/LF) while RSA did produce medium effect sizes. This could indicate that HRV parameters could be greatly influenced by speech (being largely present in the task) and its effects on respiration. This brings us to the observed underutilization of respiratory and cardiovascular dynamics parameters (e.g., RSA, BP, TWA) commonly applied in lab-based stress investigations as an important part of stress reactivity processes (e.g., respiration in relation to heart rate being essential for correct interpretation of HRV; Quigley et al., 2024; Saygin et al., 2025). Hence, we are cautious not to overinterpret the observed magnitude of effects and the underlying stress mechanisms. Instead, our results provide support for consistent and reliable induction of physiological changes in VR by various stressors and across multiple physiological measures, with subtle patterns of reactivity differentiated by type of task, stressor category, or parameter of interest. The most commonly used measures of ANS-related reactivity identified by this review, namely HR, followed by EDA and HRV parameters, showed a range of plausible effect sizes and stable stressor-induced changes (e.g., larger effects for TSST), creating trust in their applicability to VR stress induction research in general but also in the context of validation frameworks.

### Implications for wearables validation through VR stress induction

The results of our review further support the use of VR stress induction for validating wearables and the potential to integrate these, so far separate, research fields. Our findings suggest that VR meets the two prerequisites to the validation studies, as it allows us to create designs which elicit the required variations in physiology while making it possible to capture these changes with both wearables and gold-standard recording. Firstly, the stable increases in the relevant physiological parameters, combined with the consistent reactivity across different VR stress paradigms, indicate that VR can reliably produce physiological variations required for validating wearable devices on their construct validity for capturing stress responses. This is additionally supported by several studies utilizing wearable devices to successfully record physiological stress reactivity (Bolinski et al., 2021; Hirt et al., 2020; Kristensen et al., 2020; Macey et al., 2023; Morales Téllez et al., 2023; Newman et al., 2020; Schebella et al., 2020; Schulz et al., 2019; Tao et al., 2022; Weiß et al., 2020; Zhu et al., 2023). Secondly, with most studies implementing lab-based equipment, and with Schulz et al. (2019) using a wearable and gold-standard reference simultaneously, it appears that inclusion of both a wearable device and a gold-standard reference device in VR setups is highly viable. Additionally, this review provides support for utilizing VR for validating wearables on the level of measures relevant to stress reactivity. The most common and robust measures of ANS-related stress reactivity identified by this review (HR, EDA, and HRV) happen to overlap with the cardiovascular and electrodermal parameters provided by wearable devices. These measures are also in line with the current validation practices, with most existing validity frameworks not moving beyond HR, HRV, and EDA parameters (Menghini et al., 2019; Mühlen et al., 2021; van Lier et al., 2020). Considering these findings, VR offers a promising platform for controllable, repeatable induction of ANS variation for systematic and efficient assessment of wearable devices on both construct and convergent validity.

This brings us to the concept of ecological validity, and whether VR stress induction can positively influence current laboratory validation studies suffering from conditions that are often labelled as too artificial and controlled. Our findings do not offer a straightforward answer. On the one hand, in vivo stress induction was often shown to result in higher stress reactivity than that observed in VR (Ecker et al., 2024; Kelly et al., 2007; Kothgassner et al., 2021; Mostajeran et al., 2020; Schulz et al., 2019; Zimmer, Buttlar, et al., 2019a, 2019b). Such attenuation of reactivity in VR versus in vivo, while relevant if the strength of the stress response needs to be maximized, might not be a hindrance in the context of validation. When assessing the convergent validity of a wearable, the focus is on alignment of the two signals and being able to differentiate rest from a psychophysiological stress reaction. On the other hand, VR appears highly promising for creating context-rich, dynamic environments, representing real-world-like scenarios such as driving or height simulations, theoretically important for creating ecologically valid emotional experiences (Epel et al., 2018; Hoemann et al., 2020). Hence, as long as sufficient and interpretable variation in physiology is evoked, VR stressors may still be of high utility to validation protocols, particularly since they may enrich the contextual cues and characteristics of stressor elements to better approximate real-world acute stress processes.

Among the stressors identified, TSST and high-altitude tasks seem most promising for this dual purpose and are most supported by the large body of evidence for sufficient physiological reactivity. These two tasks could be used in combination in order to elicit stress through a comprehensive coverage of social-evaluative, cognitive (both present in TSST), and environmental (height task) threat. At the same time, they span multiple stressor elements, from uncontrollability, time pressure, and evaluated performance present in TSST, to physical, fear-based stressors with high unpredictability and the novelty of the high-altitude task. Such a wide coverage and combination of stressors has been shown to be beneficial for stressors outside VR as well, for example, with cognitive stressors without social evaluation not being sufficient (Dickerson & Kemeny, 2004). This is also in line with the findings of this review on Stroop. Although a similar approach was taken when designing the IMVEST task (Rodrigues et al., 2021), all the stressor elements are present concurrently. While this might be conducive to acute stress induction, we believe that separating the social-evaluative, cognitive, and environmental factors as the main source of stressor within a battery of tasks might be beneficial. New insights could be created on the potential difference, or lack thereof, between these types of stressors for physiological reactivity on various parameters. Such manipulation could be of added value to stress and affect theory, for example, in the context of disputes on the specificity of ANS responses (see, e.g., Hoemann et al., 2020).

### Need for more theoretically grounded explorations

This brings us to the observed scarcity of stress theory-based positioning of a given stressor, often being overshadowed by the pragmatic considerations. With stress theory already being highly heterogeneous, with multiple, sometimes similar and yet distinct stress models (see Epel et al., 2018, for an example of a proposed unified framework), the field of (VR) stress research would benefit from empirical work which is more clearly linked to a given theoretical basis for stress processes and which allows for interpretation of empirical results in light of the underlying theorized mechanisms. This does not necessarily mean the use of distinct stress models, but rather being deliberate with stress operationalization, measurement, stressor choice, and design, and translating both the experimental design and results to theoretically positioned reasoning. For example, while the physiological measurement of stress-related processes was quite unified between studies (although often not sufficiently theoretically substantiated), the subjective stress assessment varied greatly. This was true both for the operationalization (e.g., stress, fear, anxiety, affect all being used as subjective stress, often with no explicit explanations of the relationships between these concepts) and in the timescale and type of measurement (e.g., pre- and post-test anxiety questionnaires vs multiple repeated momentary single-item VAS assessments). The existence of such differing approaches hinders between-study comparisons and risks making the field of stress research even more convoluted, while it appears to be in urgent need of unification.

Another related issue is the observed complexity of the task design based on assumptions and with little justification in the actual findings for such design decisions. Examples here include the increased audience sizes for TSST (Delahaye et al., 2015; Santos-Ruiz et al., 2012), replicating the physical environments in VR (Shiban et al., 2016), or including physical structures in the high-altitude tasks (e.g., Liu et al., 2019; Martens et al., 2019; Meehan et al., 2003), while no clear evidence exists for their added value or even opposing evidence is present (Mostajeran et al., 2020; Zimmer, Wu, et al., 2019). The physical plank is an especially interesting example since all studies included it, making the VR setup much more complex due to the need for dedicated space, safety precautions and equipment, and seamless alignment of VR environment with physical setup, while not a single study compared the effectiveness of VR stress induction for extreme VR heights with no physical plank present. Since the utility of VR lies in its ability to create immersive virtual environments transporting subjects beyond the laboratory, a question arises as to how much we should try to connect the person back to the physical space. This is especially true with little support for the added value of such props and with the addition of a physical plank appearing counterproductive to the efficiency, safety, and ethical advantages of VR setups. Some authors additionally make strong claims about task effectiveness in different conditions, forgetting to acknowledge such essential elements as added movement or talking. All of this comes back to the need for more controlled manipulations of VR stressor elements and stress induction protocols so that our decisions can be properly justified and grounded in both theory and corresponding evidence.

### Need for standardization and more transparent reporting on VR development

Lastly, the results of our review make apparent the strong need for more detailed and transparent reporting when it comes to the content and development of the VR applications themselves, as essential parts of the experimental procedure, in order to increase reproducibility and unification of study designs. This need was concurrently recognized by other researchers, with reporting guidelines RATE–XR for VR studies emerging at the time of writing this review (Vlake et al., 2024). The authors describe the need to report the details of both the application itself and its development process—the element we have observed as the most lacking in existing literature. The lack of transparency is in turn connected to the apparent lack of standardization, with considerable heterogeneity in stressor design between studies using the same task. This highlights the need not only for more transparent and detailed reporting but, most importantly, for greater open-access availability of existing VR stress tests. With many existing solutions relying on commercial collaborations, openness of existing reusable tasks remains limited.

Most recently, the recognition of the need for open-source VR software appears to have increased. As identified by this review, the need for standardization was already addressed by the creators of IMVEST (Rodrigues et al., 2021) and the VR Stroop Room (Gradl et al., 2019). However, concurrently with writing of this review, new attempts have emerged. The most prominent example for stress research is a recently published open-source version of VR-TSST (Linnig et al., 2025), freely downloadable and evaluated on its subjective and physiological stress-inducing properties. Another relevant publication introduces an open-access VR cybersickness simulator—Cybersicker (Zielasko & Law, 2024)—a stressor representative of the *uncontrolled motion* task category identified in this review. More broadly, Rubo (2025) recently provided a tutorial on the development of multi-player social VR scenarios, accompanied by an example software that can be further adapted by other researchers to create social VR experiments. Similarly, Picard et al. (2024) introduced XR MUSE, an open-access framework for developing multi-player extended reality (XR) applications and helping to tackle issues such as networking or data synchronization. These example efforts showcase the increasing recognition of the importance of minimizing the effort required for VR development and providing easily reproducible and standardizable VR software, opening doors to more efficient and unified VR stress induction research.

### Limitations

The present review found that the field of VR stress induction shows substantial variability. This was true for study aims, VR task design, analysis methods, and outcome measures, as well as reporting practices. In line with the purpose of scoping reviews, we have focused on mapping this heterogeneity. The meaningful comparisons of tasks effectiveness were therefore limited to a qualitative, categorical, and visual synthesis. Our results suggest which specific components of established stress paradigms may be suitable for future meta-analytic synthesis. With Jackson and Turner (2017) cautioning against quantitative comparisons among fewer than five studies, it appears that the only measures that would robustly facilitate such analysis are HR, SCL, and cortisol, while α-amylase and HF could also potentially be used with the necessary caution. The large body of evidence on TSST, the common use of those three parameters, and the highly aligned design of this stressor and measurement time points and parameters make it a possible candidate for meta-analysis. In turn, the remaining evidence base on VR stress paradigms does not yet seem standardized or homogeneous enough to meaningfully support quantitative pooling. We additionally observed that studies differed greatly in sample sizes (including several pilot studies), and that sometimes claims and interpretations on task effectiveness were strapped from the stress-relevant context information—for example, where authors claim higher effectiveness achieved by novel task design when introducing additional elements that could induce higher reactivity, such as added movement, while not reflecting on those aspects (e.g., in the gamified Stroop task; Morales Téllez et al., 2023). These conclusions reflect an uneven and developing evidence base, in which both quantitative findings and qualitative insights into task design and from in vivo comparisons must be considered.

## Conclusion

This study set out to provide a comprehensive overview of VR stress induction research with a strong focus on task effectiveness and stressor design, and with additional exploration of the use of VR for wearables validation. Overall, the results of this review support the already wide use of VR for stress induction within various research contexts and across multiple stress tests identified. The high levels of subjective and especially autonomic reactivity, combined with initial support for the use of wearables, makes VR a promising platform for validating wearable devices through the use of virtual stressors. While cognitive stress tasks such as Stroop clearly might not produce the strongest physiological responses, their VR versions remain valuable for cognitive research. In turn, social-evaluative and environmental tasks categories offer a multitude of relevant stressors, with VR-TSST and the high-altitude task emerging as the two most established and proven VR stress tests. Lastly, the current breadth of approaches indicates that further standardization would be beneficial. This could be achieved by increased availability of open-access VR applications, which is currently lacking. Greater transparency in their development could also be of added value, together with more unified stressor conceptualization. Including theory-based positioning of targeted stress elements, careful manipulations, and translating the results in light of their theoretical bearing would be a valuable step towards increasing our understanding of the subjective and physiological mechanisms underlying stress reactivity. In this context, the added value of VR lies in the high control VR brings to experimental protocols, the evident potential to induce stressor-related changes, and the freedom to create various immersive, dynamic, or even more ecologically valid environments.

## Electronic supplementary material

Below is the link to the electronic supplementary material.

Supplementary file1 (XLSX 29 KB)

Supplementary file2 (XLSX 658 KB)

## Data Availability

All the relevant files (e.g., search strategy and log, extracted data) are made available as supplementary materials and will be made openly available on the OSF page upon acceptance: https://osf.io/sp5z8.

## Appendix

Tables 4 and 5

**Table 4:** Overview of ANS measures to be extracted and the most standard methods of obtaining them

Measure	Standard recording method(s)
Skin Conductance Level (SCL)	Electrodermal Activity (EDA)
non-specific Skin Conductance Responses (nsSCR)	Electrodermal Activity
Pre-ejection period (PEP)	Impedance Cardiography (ICG)
Respiration Rate (RR)	Impedance Cardiography
Blood Pressure (BP)	Oscillometry
T-Wave Amplitude (TWA)	Electrocardiography (ECG)
α-Amylase	Saliva Proteomics
Heart Rate (HR)	Electrocardiography or Photoplethysmography (PPG)
Root Mean Square of Successive Differences (RMSSD)	Electrocardiography or Photoplethysmography
High Frequency Power (HFP)	Electrocardiography or Photoplethysmography
Respiratory Sinus Arrhythmia (RSA)	Impedance Cardiography with Electrocardiography

The list is based on recommendations from de Geus and Gevonden (2024)

**Table 5:** Characteristics of studies included in this review

Study	Population	Sample size and age	VR technology*	Recording device(s)	Stress task used	Study aim	Physiological measures**	Subjective measures***
Agrawal and Vemuri (2019)	Young or inexperienced drivers	*N =*45 (28, 9.5)	HMD: Not specified	EDA sensor, not specified	Driving simulation	Exploring the effects of honking on the drivers’ behaviour and anxiety.	SCL	1-item Likert Scale
Annerstedt et al. (2013)	Healthy adults, males	*N =*30 (27.7, 6.7)	CAVE	ML866 Power Lab (ADInstruments)	TSST	Assessing stress recovery in virtual forest nature and the relevance of nature sounds.	Salivary cortisol, HR, T-wave Amp, HRV (HF, LF, LF/HF, TOT)	STAI-S (short, Y-1)
Bahr et al. (2021)	Healthy adults; males	*N =*60 (27.8, 6.9)	HMD: HTC Vive	Brain Vision Recorder (Brain Products)	TSST	Comparing the effects of the benzodiazepine alprazolam and the TSPO ligand etifoxine on HPA and SAM reactivity.	HR, SCL, salivary and blood cortisol	STAI (Y-1), VAS
Baldini et al. (2022)	Healthy adults	*N =*18 (18-35)	HMD: Oculus Rift S	Shimmer 3GSR+	Trauma/horror scenario (passive)	Developing a novel VR fear task and evaluating the associated EDA responses.	SCL, nsSCRs, (and multiple EDA features derived)	STAI (Y-1)
Ban et al. (2024)	Healthy adults	*N =*19 (20-30)	HMD: HTC Vive Pro 2	ECG sensor; PLUX biosignals	Emotional activation scenario	Investigating if scripted VR communication (customer-service) elicits HPA-axis stress and how communication load alters the response.	LF/HF, salivary cortisol	1-item Likert Scale
Baptie et al. (2021)	Healthy adults	*N =*61 (20.7, 18-52)	HMD: HTC Vive	Not used	Trauma/horror scenario (passive)	Assessing the utility of VR for Trauma Film Paradigm research by comparing the VR with an on-screen simulation on the subjective stress experience evoked.	Not used	STAI-S (short, Y-1), VAS
Biedermann et al. (2024)	Healthy adults	N = 39 (25.7, 3.7)	HMD: HTC Vive	Biopac MP150 + Bionomadix module	High altitude task (Elevated Plus Maze)	Repeated use of the task to study anxiety-related behaviour and the effect on stress of closely duplicating the real environment in VR.	HR, SCL, Respiratory Rate	STAI (Y-1), 1-item Likert Scale
Bolinski et al. (2021)	Healthy adults	*N =*41 (20.6, 3.1)	HMD: HTC Vive	Empatica E4	Virtual bar or café	Understanding the influence of immersive VR on cognitive restructuring and physiological arousal.	HR, SCL, nsSCRs	PANAS, 1-item Likert Scale
Bosse et al. (2018)	Healthy adults	*N =*52 (25.1, 7.6)	HMD: Oculus Rift	Custom made EDA sensor	Emotional activation scenario	Investigating the effect of “consequential” VR agents (seemingly able to administer an electric shock) on perceived stress.	SCL	Affect grid
Brambilla, Boccignone, Borghese, Chitti, et al. (2023)	Healthy adults	*N =*16 (24.9, NP)	HMD: Meta Quest 2	Not used	Game (active survival)	Examining the player’s stress and arousal dynamics and potential estimation of such variation throughout the gaming experience.	Not used	
Brambilla, Boccignone, Borghese, Croci, et al. (2023)	Healthy adults	*N =*13, (18-29)	HMD: Meta Quest 2	Not used	Game (active survival)	Assessing the Stressful Experience (StrEx) application (horror game) which adapts content based on participants’ stress levels.	Not used	Dante, VAS
Breuninger et al. (2017)	Panic disorder/agoraphobia and healthy controls	48 Patients: (43.4, 12.1) Controls: (22.7, 4.4)	HMD: Not specified	Varioport-II portable device; Becker-Meditec	Emergency simulation (active)	Comparing VR emergency-stressor responses in panic patients (agoraphobic) and controls, focusing on psychophysiology, interoception, and emotion regulation.	HR, HF, SCL	4-item Likert Scale
Bu (2020)	Healthy adults; males	*N =*9 (19-22)	HMD: HTC Vive	WEB-1000; Nihon Kohden	(Un)controlled movement (roller coaster)	Developing a VR roller-coaster mental stress test and examining physiological stress responses.	RR interval, SDNN, Distance Poincare index (´Dis)	Not used
Bullinger et al. (2005)	Healthy adults	*N =*94 (46.4, 14.3)	HMD: Virtual Research	salivary cortisol only	Cognitive task (other)	Testing whether dynamic versus static VR with or without cognitive load elicits cortisol responses and gender differences.	salivary cortisol	Not used
Cho et al. (2017)	Healthy adults	12 (27.5, 3.2)	HMD: Gear VR	Biopac MP150; with PPG and EDA	Cognitive task (other)	Classifying stress levels during five phases of varied cognitive load using PPG, EDA and skin temperature features and assessing their concordance with intended stress levels.	HR, RR interval, SDNN, SDSD, RMSSD, pNN20, pNN50, LF, HF, LF/HF, ApEn, SampEn, based on Poincare plot (SD1, SD2, and SD1/SD2), for EDA: SCavg, SCL, SCL slope, SCR avg, max and number of peaks; skin temp avg, slope and std	STAI (Y-1), VAS
Counotte et al. (2018)	Psychosis patients, those at high risk, siblings, and healthy controls	123	HMD: Not specified	Not used	Virtual bar or café	Investigating the interplay between childhood trauma, immune cell profiles, and response to VR social stress in psychosis.	Not used	VAS, SUD
Daviaux et al. (2020)	Healthy adults; females	*N =*12 (28, 9.5)	Driving simulator (real car repurposed; with 230° panoramic display)	Biopac MP36 with EDA	Driving simulation	Simulation of stressful driving events and its potential to be reliably measured by EDA.	ampSCR	VAS
Delahaye et al. (2015)	Healthy adults	*N =*31 (25.1, 6.6)	CAVE: 3D Powerwall	BrainAmp ExG; Brain Products	TSST; Cognitive task (other)	Examining how stress in a realistic VR emergency affects cognition and decision-making components.	HR	3-item Likert Scale
Diemer et al. (2016)	Acrophobia and healthy controls	*N =*80 Patients: (37.9, 13.9) Controls: (33.7, 11.9)	HMD: Z800 3D Visor, eMagin	V-AMP; Brain Products	High altitude task	Testing whether a VR height challenge elicits physiological and subjective fear responses in acrophobia patients and if those reactions differ for controls.	salivary cortisol, HR, SCL	VAS
Dietz et al. (2022)	Healthy adults	*N =*15 (31.9, 13.9)	HMD: Valve Index headset	BITalino; Plux Biosignals	High altitude task	Comparing VR balance-training strategies under stressful height conditions and evaluating balance performance and stress markers.	SCL, nsSCR(s), HR,	NA
Domes and Zimmer (2019)	Healthy adults; males	*N =*43 (18-50)	Not reported	Not used, only saliva sampling	TSST	Investigating the effects of acute psychosocial stress on the ability to detect facial emotions.	Cortisol (Salivary), Alpha amylase	VAS
Donahue and Shrestha (2019)	Healthy adults	*N =*34 (25.6, 9.6)	HTC Vive	Not used	Stroop	Developing and validating a VR office task to assess response inhibition under stress.	Not used	DTS, PANAS
Dong et al. (2020)	Healthy adults	*N =*60 No age information	Not reported	Not used	Emergency simulation (active)	Estimating gender differences in stress levels and sign searching behaviour in a virtual fire emergency.	Not used	11-item Likert Scale (also about task design)
Ecker et al. (2024)	Healthy adolescents	*N =*73 (13.9, 1.9)	HMD: HTC Vive Pro Eye	EcgMove 4; Movisense	TSST	Validating a VR version of the TSST-C and comparing it with the real TSST-C.	HR, RMSSD, Cortisol (Salivary), Alpha Amylase	STAI (Y-1), VAS
Eftekharifar et al. (2021)	Healthy adults	*N =*22 (25.4, 4.1)	HMD: HTC Vive Pro	ProComp Infiniti; ThoughtTechnology	Cognitive task (other; mental arithmetic)	Comparing restorative effects of VR nature when being surrounded by the scene versus viewing it form an enclosed virtual room as a flat picture.	Just EDA mentioned, from text and values it was most likely SCL	ZIPERS
Eudave and Valencia (2017)	Healthy adults; males	*N =*5 (31.2, 4.6)	HMD: Oculus Rift CV1	BITalino; Plux Biosignals	Driving simulation	Exploring presence and physiological responses during a VR driving task while wearing an HMD.	HR, EDA (in arbitrary units instead of μS, values around 920)	Not used
Fich et al. (2014)	Healthy adults; males	*N =*30 (23.9, 19-31)	CAVE	ML866 Power Lab; ADInstruments	TSST	Investigating whether indoor architectural design can influence stress responses.	HR, HF, TWA, salivary cortisol	Not used
Finseth et al. (2018)	Healthy adults	*N =*13 (23.9, 3.6)	HMD: HTC Vive	Biopac ECG (no further details)	High altitude task	Testing game-based stressors in a high-altitude simulation to reliably elicit stress reactivity.	HR, salivary cortisol	Not used
García-León et al. (2019)	Healthy adults	*N =*80 (21.1, 4.2)	Not reported	Not used; only saliva sampling	TSST	Evaluating how resilience relates to perceived, chronic and cortisol-based stress and psychopathology in healthy adults.	salivary cortisol, hair cortisol (to evaluate chronic stress)	PSS (only pre-stressor)
Geraets et al. (2018)	Psychosis patients, those at high risk, siblings, and healthy controls	*N =*156 For healthy controls (24.3, 4.3), all groups similar	HMD: Sony HMZ-T1	Not used	Virtual bar or café	Investigating interpersonal distance in VR social stress environments for different psychosis-liability groups and its relation to symptoms and mental states.	Not used	VAS
Giron et al. (2023)	Healthy adults	*N =*27 (24, 2.1)	HMD: HTC Vive	g.USBamp, g.GSR box; g.Tec	Social-evaluative (other; job interview)	Using a VR job interview with semi-automated agents to induce continuous everyday-life stress and derive a dynamic physiological stress index.	sSCR(s) (event based), HR, Respiration Rate	1-item Likert Scale
Gradl et al. (2019)	Healthy adults	*N =*32 (23.3, 3.5)	HMD: HTC Vive	NeXus-10 MKII; MindMedia	Stroop; Stroop Room	Evaluating a novel VR version of Stroop, the Stroop Room (Stroop supplemented with environmental elements), as a method for stress elicitation.	SCL, nsSCR(s), HR, Alpha-amylase	SSSQ
Halbeisen et al. (2023)	Healthy adults	*N =*43 (24.4, 19-47)	HMD: Oculus Rift DK2	Nellcor OxiMax DS100A; Medtronic	TSST	Examining how ethnic context in a VR-TSST modulates endocrine stress responses.	salivary Cortisol, HR, SCL, salivary Alpha Amylase	VAS
Hanshans et al. (2024)	Healthy adults	*N =*20 (26, 21-60)	HMD: Oculus Quest 2	Biopac MP36	Trauma/horror scenario (passive)	Displaying acute stress in real time as biofeedback in VR to enable participants to regulate their stress levels.	HR, HRV, SCL, Skin temperature, EEG	Self-made stress and experience items
Hartanto et al. (2014)	Healthy adults	*N =*16 (22.4, 2.4)	HMD: eMagin Z800	Mobi8; TMSi systems	Virtual bar or cafe	Inducing anxiety by manipulating VR dialogue feedback and assessing its usefulness for exposure therapy.	HR	SUD
Heffer et al. (2023)	Healthy adults	*N =*52 No age information	HMD: HTC Vive	Not used	Trauma/horror scenario (passive)	Investigating the relationship between multisensory processing of emotional stimuli and the development of intrusive memories following exposure to a VR analogue trauma.	Not used	VAS
Heming et al. (2024)	Healthy adults; medical students	*N =*54 (22.2, 2.1)	HMD: Oculus Rift S	OnTrak; Spacelabs	TSST	Examining whether hair cortisol concentration predicts acute stress responses and recovery to VR-TSST in medical students.	HRV (RMSSD), BP	Not used
Hirt et al. (2020)	Healthy adults	*N =*27 (25.4, 5.9)	HMD: HTC Vive with an integrated eye tracker from Pupil Labs (@200 Hz)	A pulse chest belt linked to a Garmin Edge 1000	Emergency simulation (active)	Assessing stress levels during VR exposure to an emergency simulation using eye tracking and pulse.	HR, pupil dilation	Other
Isnanda et al. (2014)	Healthy adults; university students	*N =*24 (28.4, 4.8)	HMD: Sony HMZ-T2 Personal 3D Viewer	Mobi8; TMSi systems	Virtual bar or café	Examining how the stream of paranoia-evoking events in a VR restaurant affects paranoid ideation.	HR, SCL	SUD - VAS, other
Jönsson et al. (2010)	Healthy adults; males	*N =*10 (28.3, 24-38)	CAVE	Not used; only saliva sampling	TSST	Assessing if a VR-TSST (simulated via CAVE) induces and habituates stress responses similarly to the previously established effects of the real TSST.	HR, T-Wave Amplitude, HF-HRV, Cortisol (Saliva)	STAI (Y-1)
Jönsson et al. (2015)	Work-stress related exhaustion disorder and healthy controls	*N =*51 (48.7, 7.1)	Not reported	ML866 Power Lab; ADInstruments	TSST	Examining dysfunctional flexibility of HPA and SAM responses and ANS reactivity to VR-TSST in early and recovered eating disorders.	Cortisol, Alpha amylase, HR, RR, TWA	STAI (Not specified further)
Kamińska et al. (2021)	Healthy adults	*N =*28 (36.5, 10.4)	HMD: Oculus Quest	EpocFlex; EMOTIV	Stroop; office setting	Investigating the use of EEG signals to classify a subject’s stress level while using a VR environment and assessing the subjective stress levels.	Theta, Alpha, Gamma, Beta brain waves	Questionnaires unspecified (current stress levels and mood)
Kelly et al. (2007)	Healthy adults	*N =*274 (21.5, 5.3)	Not reported	Not used; only saliva sampling	TSST	Determining whether a VR-TSST with a virtual audience elicits cortisol responses comparable to a real audience.	salivary cortisol	Likert
Kerous et al. (2020a)	Healthy adults	*N =*16 No age information	HMD: HTC Vive	Psychlab data acquisition system	Stroop; Emotional and social	Finding an optimal combination of social and task-related stressors to reliably induce stress.	nSCR, SCL, HR, RMSSD	Not used
Kerous et al. (2020b)	Healthy adults	*N =*39 (33.0, 9.4)	HMD: HTC Vive	Psychlab data acquisition system	Stroop; Emotional and social	Testing whether a VR social environment elicits measurable stress, and identifying suitable physiological measures and predictors.	nSCR, SCL, HR, RMSSD	SAS
Kothgassner, Felnhofer, et al. (2016a, 2016b)	Healthy adults	*N =*66 (24.2, 3.0)	HMD: eMagin Z800 3D, and two stereoscopic SVGA displays	portable M-EXG; Schuhfried	Social-evaluative (other)	Comparing stress responses to public speaking in front of (1) real, (2) virtual audiences, and (3) an empty VR hall.	HR, rRMSSD, salivary cortisol	STAI
Kothgassner, Hlavacs, et al. (2016)	Healthy adults	*N =*13 (22-26)	HMD: Sony HMZ-T1	Not used; only saliva sampling	TSST and Cognitive (other; Cyberball)	Creating and validating VR versions of Cyberball and TSST as ecologically valid social stress paradigms.	salivary cortisol	VAS
Kothgassner et al. (2021)	Healthy adults	*N =*68 (24.1, 2.7)	HMD; Sony HMZ-T1	portable M-EXG; Schuhfried	TSST	Comparing physiological reactivity and habituation across repeated in-vivo and VR-TSST exposures.	Cortisol, HR, HRV	FNE, VAS
Kotlyar et al. (2008)	Healthy adults	*N =*12 (30.2, 11.6)	Not reported	enzyme immunoassay kit; Diagnostic Systems Laboratories	Social-evaluative (other)	Determining whether VR public speaking induces physiological stress.	Cortisol, HR, BP, epinephrine, norepinephrine	STAI
Kristensen et al. (2020)	Healthy adults	*N =*21 (22, 19-26)	HMD: HTC Vive	Wearable: Polar H10	Cognitive task (other)	Comparing post-VR-stressor relaxation from viewing art and nature with music in VR versus on a TV screen.	HR, pNN50, RMSSD, HF power, LF/HF ratio	RRS
Laskowitz and Huffstadt (2023)	Not reported	*N =*10 (27, 18-35)	Not reported	Not used	TSST	Examining how self-similar versus desirable avatars in stressful VR situations affect self-presence and self-awareness.	Not used	Likert scale
Lebamovski and Gospodinova (2023),	Not reported	*N =*5 No age information	HMD: Photontree 3D Pro helmet	ECG recording, details not provided	(Un)controlled movement (flying)	Evaluating a VR flying scenario on its stressful effects.	LF, HF, LF/HF	Not used
Lee et al. (2023)	Psychosis patients and healthy controls	*N =*126 (40.2, 10.4)	HMD; Oculus Rift CV1	earlobe PPG; FNIKorea	TSST	Investigating physiological responses to a VR-TSST in patients with psychosis versus healthy controls.	HR, SCL, Cortisol (blood), RR	PANAS
Legkov et al. (2017)	Healthy adults	*N =*22 (23.8, SD not provided)	HMD: Oculus Rift DK2	Arduino-based E-Health Shield for EDA	Cognitive task (other; navigation and shooting)	Inducing cognitive stress by increasing cognitive load through a dual task in VR, and quantify the impact of cognitive stress on path integration.	Tonic and Phasic SCL	SSSQ
Lin et al. (2020)	Healthy adults	*N =*9 (19-24)	HMD: HTC vive helmet	Unclear, likely wearable: movement bracelet of Zhongwei Digital Company	Emergency simulation (active)	Exploring physiological stress reactions of subway passengers in VR fire scenarios to inform stress management practices.	HR, BP	Not used
Linninge et al. (2018)	Healthy adults (High-low Burnout)	*N =*40 (33.2, 9.5)	CAVE	ML866 Power Lab; ADInstruments	TSST	Examining intestinal permeability and physiological and inflammatory markers of reactivity to acute psychosocial stress in high-stressed and low-stressed individuals using a virtual version of the TSST	HR, HF, Cortisol (saliva and blood), zonulin and multiple inflammatory markers	STAI
Liszio and Masuch (2019)	Healthy adults	*N =*44 (23.7, 5.67)	HMD: Oculus Rift	Not used	TSST	Investigating whether interactivity enhances the relaxing and mood-enhancing effects of natural VR environments.	HRV	PANAS, STAI, SSQ
Liu et al. (2019)	Healthy adults; males	*N =*44 (23.6, 2.9)	HMD: HTC Vive Pro	Not used; only saliva sampling	High altitude task	Evaluating a high-altitude task for inducing stress and assessing cognitive performance under stress.	salivary cortisol	Likert, PANAS
Liu and Zhang (2020)	Healthy adults	*N =*57 (23.3, 2.1)	HMD: nVisor SX60	Biopac MP150	TSST	Examining VR-TSST effects with a placebo control and assessing sex differences in psychophysiological responses.	HR, SCL, LF, HF, LF/HF	VAS, BAI, PANAS, SCSQ
Lugrin et al. (2016)	Healthy adults	*N =*38 (21.1, 2.0)	HMD: Not specified	Not used	High altitude task	Investigating how avatar anthropomorphism influences fear of heights in immersive VR.	SCL	Other
Macey et al. (2023)	Healthy adults	*N =*61 (32, 8.1)	HMD: Oculus Quest 2	Wearable: Empatica E4	TSST	Investigating how perceived tallness versus perceived shortness, without any visible virtual body or representation, influences state speech anxiety and emotional responses to a stressful speech task.	HR, nsSCRs, nsSCRs amplitude	PSAS, SAM
Martens et al. (2019)	Healthy adults; males	*N =*28 (24.9, 1.5)	HMD: nVisor SX111	SC-EKG; Psychlab	High altitude task	Assessing whether a stressful VR scenario elicits sympathetic stress responses and impacts memory performance.	HR, salivary cortisol, alpha-amylase, SCL	VAS, other
McAllister et al. (2024)	Healthy students	*N =*53 (21, 3)	HMD: HTC Vive Pro	Wearable: Polar Verity Sense	Game (active survival)	Examining the impact of caffeine on subjective and physiological stress, as well as performance during a virtual shooter game.	HR, salivary cortisol, alpha amylase, Immunoglobin A	STAI (Y-1)
Meehan et al. (2003)	Healthy adults	*N =*164 (35, 10.9)	HMD: V8	ProComp+; Thought Technology	High altitude task	Testing whether higher VR realism (lower VR system latency) increases presence, physiological stress responses and reduces simulator sickness	HR, SCL	Single-item from UCL Behavioral Presence Questionnaire
Mevlevioğlu et al. (2022)	Not reported	*N =*2 No age information	HMD: HTC Vive and Oculus Rift S	Brainband; Myndplay, Shimmer GSR; Shimmer	Stroop; emotional	Eliciting different stress levels with VR Stroop and recording brain, heart and electrodermal activity.	SCL, HR, EEG	VAS
Misztal et al. (2020)	Healthy adults	*N =*6 (24.2, 22-28)	HMD: Oculus Quest	Not used	Game (active survival)	Investigating whether added visual effects intensify perceived threat and stress and affect VR experience quality.	Not used	1-item Likert scale
Montero-López et al. (2016)	Healthy adults; females	*N =*45 (21.2, 3.8)	HMD: eMagin Z800 3DVisor	EDA recording not specified, saliva sampling	TSST	Comparing the VR and on-screen TSST protocols on sympathetic and HPA activation and exploring sex differences.	SCL, Cortisol	PSS
Montero-López et al. (2018)	Healthy adults; females	*N =*42 (33.6, 7.8)	HMD: not specified	Only saliva sampling	TSST	Investigating how menstrual cycle phase relates to daily cortisol patterns and VR-TSST cortisol responses.	salivary cortisol	Not used
Morales Téllez et al. (2023)	Healthy adults	*N =*27 (26, 3.61)	HMD: HTC Vive Pro	Wearable: Polar H10	Stroop	Comparison of VR Color-Word Stroop Test and its gamified version to in-vivo.	HR	DASS-21 (stress scale)
Mostajeran et al. (2020)	Healthy adults	*N =*24 (28.2, 7.5)	HMD: HTC Vive	medilog® AR12 plus ECG; SCHILLER, NUL GSR; NeuLog	TSST	Comparing in-vivo and VR TSST and testing the effects of different virtual audience sizes on stress reactivity.	HR, SCL, salivary Cortisol	STAI, SAM, VAS, SSA
Newman et al. (2020)	Healthy adults	*N =*58 (21, 5.6)	HMD: Oculus Rift	Wearable: Polar H10	High altitude task	Examining how VR heights affect executive function and how this is modulated by negative appraisals of heights.	HR, RMSSD	Likert
Nouri et al. (2022)	Healthy adults	*N =*126 (26.3, 18.4-35)	HMD: HTC Vive	Biopac MP150	High altitude task	Relating circulating estradiol levels to behavioural, subjective and physiological indicators of anxiety.	HR, SCL, salivary Cortisol	STAI, Likert
Ostermeier and Schmoll (2022)	Healthy adults	*N =*10 (26.3, 3.9)	HMD: Oculus Go	Bio Amp FE231; ADInstruments	(Un)controlled movement (roller coaster)	Comparing different strategies for increasing the sympathetic activity without exercise, by analysing the heart rate as affected by the VR roller coaster.	HR	Not used
Pallavicini et al. (2013)	Healthy adults	*N =*39 (21.2, 3.6)	HMD: Vuzix iWear VR920	NeXus-4; MindMedia	Social-evaluative (other)	Investigated if technological breakdowns during VR could reduce its effectiveness in comparison with other media (text, audio, and video) in arousing emotions related to a real-life stressor.	RR, HF, EMG_RMS (Root Mean Square EMG)	Other
Park et al. (2020)	Healthy adults	*N =*32 (27.8, 4.9)	HMD: Oculus Rift	Biopac MP150; Biopac	Trauma/horror scenario (passive)	Investigating how audio presentation, visual reproduction mode (VR vs on-screen), and scene type affect psychological and physiological responses.	fEMG, HR, RR, SCL	Other
Phillips et al. (2010)	Healthy adults	*N =*31 (21.6, 4.1)	HMD: nVisor SX	EKG-Flex/Pro and SC-Flex/Pro; Thought Technology	High altitude task	Determining reliable presence measures and comparing them across immersive virtual environments with different properties.	HR, SCL	Likert
Phillips et al. (2012)	Healthy adults	*N =*33 (21.6, 4.1)	HMD: nVisor SX	EKG-Flex/Pro and SC-Flex/Pro; Thought Technology	High altitude task	Comparing presence levels evoked by three immersive VR height conditions previously shown to differ in distance perception.	HR, SCL	PQ, SUS
Poguntke et al. (2019)	Healthy adults	*N =*15 (23.5, 2.5)	HMD: HTC Vive	NeXus-4; MindMedia	Stroop; social	Investigating how a classic Stroop stress task transfers to immersive VR and how stress is perceived and measured.	HR, HRV (as provided by the device's algorithm)	SSSQ
Pot-Kolder et al. (2018)	Psychosis (four high-to-low liability groups)	*N =*170 (25.5, 4.6)	HMD: Sony HMZ-T1	Not used	Virtual bar or café	Manipulating VR social stressors to test how cognitive biases moderate paranoid ideation across psychosis-liability groups.	Not used	Paranoid ideation
Pratiher et al. (2022)	Healthy adults	*N =*31 (25, 3.2)	HMD: HTC Vive Pro	Biopac MP160	Game (active survival)	Analysing HRV metrics across VR shooting difficulty levels and timescales to characterise stress-related reactivity.	HFpeak, HF, LF, VLF (and nonlinear HRV parameters based on the Poincaré plot)	Not used
Rodrigues et al. (2020)	High Trait Anxiety; males	*N =*107 (20.5, 2.2)	HMD: HTC Vive	Biopac MP150	Cognitive task (other; maze)	Developing immersive virtual environments to predict individual physiological stress susceptibility.	HR, RR, ECG, RMSSD	STAI
Rodrigues et al. (2021)	Healthy adults; males	*N =*118 (20.6, 2.1)	HMD: HTC Vive	Biopac Bionomadix	Cognitive task (other; IMVEST)	Presenting and validating IMVEST, an open-access, adaptive, multimodal VR stress test.	sCortisol, Respiration Rate, HR, LF/HF, HRV (Triangular index), Heart deceleration capacity, nsSCRs	NA
Sabo et al. (2018)	Healthy adults	*N =*12 No age information	HMD: HTC Vive	FAROS 90° ECG; EDA Consensys; Shimmer	(Un)controlled movement (roller coaster)	Establishing a methodology for creating a database of speech recorded under stress.	HR, EDA (skin resistance) - SCL	SACL
Sahoo et al. (2021)	Healthy adults	*N =*31 (25, 3.2)	Not reported	EEG cap; Biosemi ActiveTwo System	Game (active survival)	Exploring EEG power-spectrum changes during VR shooting tasks with varying difficulty and across sessions.	EEG: Absolute Average Band Power (ABP) features and Relative average Band Power (RBP) for different frequency bands (Delta, Theta, Alpha, Low Beta, High Beta, Gamma)	Not used
Santos-Ruiz et al. (2012)	Healthy adults; females	*N =*38 (28, 11.1)	Not reported	Saliva sampling	TSST	Testing whether women with better decision-making skills experience less stress during public speaking.	salivary Cortisol	PANAS
Sanz et al. (2015)	Healthy adults	*N =*18 (25.6, 0.9)	CAVE: ARTrack	ECG and EDA sensors; g.Tec	Game (active survival)	Developing a VR game-based training scenario that manipulates feedback to induce variations in performance, behaviour and stress levels.	average RR intervals, StD of RR intervals, HRV index, LF, HF, SCL, nsSCRs	Likert
Schebella et al. (2020)	Healthy adults	*N =*52 (37.6, 10.6)	HMD: Oculus Rift	Wearable: Empatica E4	TSST	Testing how biodiversity level (urban, low, moderate and high) and multisensory cues (visual, auditory and olfactory) influence recovery from acute stress.	HR,	VAS
Schote et al. (2022)	Primary focal hyperhidrosis (PFH) patients	*N =*27 (34.4, 16.5)	HMD: Oculus Rift DK2	mobile ECG device; Neurocor	TSST	Comparing PFH patients and healthy controls on subjective, endocrine and autonomic stress reactivity.	HR, Cortisol, sAA, Axillary sweat secretion	VAS
Schröder and Mühlberger (2023)	Smokers and non-smokers	*N =*71 (22.6, 3.7)	HMD: HTC Vive	Not used	TSST	Investigating the influence of psychosocial stress on attentional bias (AB) towards smoking-related stimuli among smokers relative to non-smokers	Not used	VAS
Schudy et al. (2023)	Healthy adults	*N =*6 (28, 1.5)	HMD: HTC Vive Pro	Not used	Social-evaluative (other; Metro Stress Task (VR-MST)	Presenting the design and preliminary evaluation of the VR Metro Stress Task (VR-MST).	NA	Other
Schulz et al. (2019)	Healthy adults	*N =*28 (30.7, 10.6)	HMD: HTC Vive	BITalino; Plux Biosignals and a wearable chest strap	High altitude task	Comparing presence and stress in real climbing versus VR climbing with and without physical prop	nsSCRs, HR, average RR intervals	VAS
Schweizer et al. (2017)	High Trait Anxiety	*N =*80 (21.9, 4.1)	HMD: TriVisio VR Vision	Varioport-II; Becker-Meditec	Emergency simulation (active)	Examining how high trait anxiety influences affective and cognitive processing in a VR analogue trauma paradigm.	SCL	Likert
Schweizer et al. (2019)	Healthy adults	*N =*80 (21.7, 2.3)	HMD: TriVisio VR Vision	Varioport-II; Becker-Meditec	Emergency simulation (active)	Investigating cognitive and psychophysiological regulatory mechanisms that shape analogue trauma symptoms in real time, as relevant to PTSD.	SCL, HR,	BAI, other
Seinfeld et al. (2015)	Healthy adults	*N =*40 (26.3, 3.7)	CAVE: Event Lab, CrystalEyes4 glasses	NeXus-4; MindMedia, ECG; Mobilab	High altitude task	Comparing VR height exposure with and without relaxing background music on stress levels and behaviour.	SCL, HR,	STAI, SUD - VAS, other
Shiban et al. (2016)	Healthy adults; males	*N =*45 (23.8, 2.5)	HMD: nVisor SX	V-AMP; Brain Products	TSST	Testing if a virtual competitor increases stress reactions in VR-TSST and comparing VR and in-vivo TSST responses.	SCL, HR, salivary cortisol	VAS
Skarbez et al. (2018)	Healthy adults	*N =*32 (19.5)	HMD: nVisor SX	ProComp Infiniti System; Thought Technology	High altitude task	Investigating how manipulating immersion and presence components in a VR visual cliff affects virtual experience and stress responses.	E1: HR, mean RR interval, SCL, LF, HF, LF/HF	Not used
Spangler et al. (2021)	Healthy adults	*N =*17 (26.2, 3.7)	HMD: HTC Vive	Active2; Biosemi, Finapres portable device;Finapres	Game (active survival)	Examining performance and cardiovascular physiology over time during repeated VR shooting tasks.	RMSSD, HR, SDNN, SBP, DBP	Not used
Tao et al. (2022)	Healthy adults	*N =*57 (20.9, 1.9)	HMD: HTC Vive Pro	Wearables: chest strap (BodyPlus) and wrist band (B4RealTime)	Game (active survival)	Evaluating a novel VR stress simulation system and investigating the relation between physiological data and self-reported stress ratings.	HR, SCL	SSAI, other
Turner et al. (1997)	Healthy adults; males	*N =*11 (24.2, 1.5)	HMD: Kaiser-Optic Visual Immersion Monitor (VIM 500)	NCCOM-3 Model 6, Bo-Med Medical Manufacturing, Ltd., Irvine, CA; Dinamap Adult/Pediatric Vital Signs Monitor	Driving simulation	Assessing the task’s usefulness for future lab-based stress research by examining its ability to elicit hemodynamic responses, test–retest reliability, perceived realism, and gender differences in responses.	systolic blood pressure, diastolic blood pressure, cardiac output, and calculated total peripheral resistance	Not used
Van Dammen et al. (2021)	Healthy adults	*N =*18 (24.6, 6.9)	HMD: HTC Vive	portable ECG; Mindware Technologies	Social-evaluative (other; dance)	Testing whether a VR dance-competition task with social evaluative threat elicits physiological responses.	HR, Respiratory Sinus Arrhythmia (RSA,) Cortisol (Salivary), Testosterone	Not used
Vatheuer et al. (2021)	Healthy adults; males	*N =*74 (24.9, 5)	HMD: Oculus Rift DK2	Only saliva sampling	TSST	Investigating gaze behaviour under acute social stress in males with varying social anxiety levels	salivary Cortisol	Other
Veling, Counotte, et al. (2016a, 2016b)	Psychosis	*N =*170 (25.4, 4.7)	HMD: Sony HMZ-T1	Not used	Virtual bar or café	Testing links between childhood trauma, social stress sensitivity, psychotic and affective symptoms and psychosis liability using VR.	NA	SUD - VAS, other
Veling, Pot-Kolder, et al. (2016a, 2016b)	Psychosis (different liability groups)	*N =*170 (25.4, 4.6)	HMD: Sony HMZ-T1	Not used	Virtual bar or café	Testing social stress sensitivity as a mechanism of psychosis, by exposing individuals with different levels of liability to psychosis to 5 social stress environments in VR.	NA	GPTS, SIAS, CAPE, SUD, SSPS
Verdejo-Garcia et al. (2015)	Excess weight	*N =*81 (15.6, 1.9)	Not reported	Only saliva sampling	TSST	Examining if adolescents with excess weight are more sensitive to social stress and hence to harmful effects of stress in cognition.	Cortisol	VAS
Wang et al. (2009)	Healthy adults	*N =*735 (17.8, 3.2)	HMD: Kaiser-Optic Visual Immersion Monitor (VIM 500)	BioZ; Cardio-Dynamics	Driving simulation	Determining whether the genetic influences on HRV under stress are different from those at rest and the extent to which they depend on ethnicity or gender.	RMSSD, HF	Not used
Weber et al. (2024)	Healthy adults	*N =*60 (22.3, 2.1)	Not reported	eMotion Faros 180◦; Mega Electronics	TSST	Testing whether Perceived Stress Reactivity Scale (PSRS) scores predict cardiovascular and psychological responses to daily-life and VR-TSST stress.	HR, HF, LF, DBP, SBP	PSRS, Other
Weiß et al. (2020)	Healthy adults	*N =*14 80%<35	HMD: HTC Vive Pro	Grove GSR sensor; SEEED, heart-rate monitor; Zephyr	High altitude task	Evaluating whether integrated non-invasive sensors and self-estimated stress levels and sensor outputs align in reflecting stress responses.	SCL, HR, RR	SSSQ, other
Weiß et al. (2022)	Healthy adults	*N =*12 (26, 8)	HMD: HTC Vive Pro Eye	Bioharness; Zephyr	Emergency simulation (active)	Exploring how multimodal sensory presentation in virtual reality can be used to elevate stress levels and evaluating its effectiveness for stress induction.	HR, RR	Semi-structured qualitative interviews
Yin et al. (2020)	Healthy adults	*N =*100 (29, 12)	HMD: HTC Vive	EdaMove3 and EcgMove3; Movisens, Omron EVOLV; Omron Healthcare	Cognitive task (other)	Evaluate the restorative effects of VR environments (offices with varying biophilic elements) on acute stress recovery.	HR, HRV, SCL, Blood pressure (SBP, DBP)	STAI-S (short, Y-1)
Zhu et al. (2023)	Healthy adults	*N =*20 (26, 4.7)	HMD: HTC Vive Pro	Bioharness; Zephyr	High altitude task	Exploring how an elevated physical platform with different virtual heights affects stress during VR height exposure.	HR, HRV (SDNN), SCL	DASS-8
Zimmer, Wu, et al. (2019)	Healthy adults; males	*N =*50 (24.8, 4)	HMD: Oculus Rift DK2	mobile ECG device; Neurocor	TSST	Testing if the similarity of the real and virtual world (virtual lab replica) modulates the stress response during a virtual TSST by intensifying the experience of presence.	salivary cortisol, alpha amylase, HR,	VAS
Zimmer, Buttlar, et al. (2019a, 2019b)	Healthy adults; males	*N =*86 (24.5, 4.3)	HMD: Oculus Rift DK2	Varioport; Becker Meditec	TSST	Comparing in-vivo and VR-TSST (together with placebo conditions) on elicitation of psychophysiological stress.	salivary cortisol, salivary alpha amylase (sAA), HR, SCL	VAS

*HMD – Head Mounted Display, CAVE – Cave Automatic Virtual Environment (stereoscopic projection room) **HR – Heart Rate, T-wave amp – T-wave Amplitude, HRV – Heart Rate Variability (HF – High Frequency Power, LF - Low Frequency Power, LF/HF – Low Frequency – High Frequency Power Ratio, SCL – Skin Conductance Level, nsSCRs – non-specific Skin Conductance Responses, ampSCRs – Amplitude of Skin Conductance Responses ***STAI – State-Trait Anxiety Inventory (Spielberger et al., 1983), PANAS - The Positive and Negative Affect Schedule (Watson et al., 1988), DASS-21 - The Depression Anxiety Stress Scale (Parkitny & McAuley, 2010)

## Declarations

### Open practices statement

The protocol for this review was preregistered at OSF: https://osf.io/sp5z8 – all other relevant materials will be made available as supplementary and on the project’s page upon acceptance.
